# Feasibility study of coke combustion and energy exergy analysis in the De-Coke process of cracking furnaces in olefin unit

**DOI:** 10.1038/s41598-025-16305-w

**Published:** 2025-08-20

**Authors:** Mostafa Moshtagh, Ahmad Azari, Marziyeh Hoseinpour, Rouhollah Fatehi, Farhad Ghadyanlou, Rahim Karami, Mohammad Rasul, Mohammad Akrami

**Affiliations:** 1 Faculty of Petroleum, Gas and Petrochemical Engineering, Persian Gulf University, P.O. Box 75169-13817, Bushehr, Iran; 2 Applied Computational Fluid Dynamics Research Group, Oil and Gas Research Center, Persian Gulf University, Bushehr, 75169 Iran; 3 Department of Biosystem Engineering, Faculty of Agriculture, Ferdowsi University of Mashhad, Mashhad, Iran; 4 Department of Mechanical Engineering, School of Engineering, Persian Gulf University, Bushehr, 75169 Iran; 5 School of Engineering, Newcastle University, Callaghan, Australia; 6 Fuel and Energy Research Group, School of Engineering and Technology, Central Queensland University, Rockhampton, QLD 4702 Australia; 7 Department of Engineering, University of Exeter, Exeter, EX4 4QF UK

**Keywords:** Combustion, Cracking, De-Coke process, Numerical analysis, Energy-exergy analysis, Mechanical engineering, Energy science and technology

## Abstract

This study investigates the numerical simulation of cracking furnaces and the feasibility of coke combustion in the De-Coke flow, utilizing computational fluid dynamics (CFD) and energy-exergy analysis. Employing the Euler-Lagrange approach, we simulate the motion of coke particles within the model. A turbulent model is applied to assess the combustion processes, while non-premixed models simulate fuel and coke particle interactions. Additionally, we incorporate the Discrete Ordinates Model for radiation and the Discrete Phase Model for coke particle motion simulation. Results indicate that injecting coke particles with dry air leads to a 100% conversion rate. However, increasing the temperature of the De-Coke stream from 454 K to 654 K yields only a slight increase in coke conversion from 52 to 55%, suggesting that sufficient time and temperature are crucial for complete combustion. The energy and exergy efficiency of the combustion furnace during the cracking process stand at 44.8% and 29%, respectively, compared to 93.24% and 96.2% during the coil cracking process. Furthermore, the destruction exergy for the combustion furnace is approximately 36%, whereas the coil experiences destruction exergy of less than 4%. Although energy and exergy distributions reveal similar trends for both conventional and burning-coke De-Coke processes at the coil, the burning-coke method offers increased destruction exergy and enhanced heat transfer, albeit at the cost of efficiency in energy and exergy transfer to the coil compared to conventional methods.

## Introduction

Refineries and petrochemicals are industries that use widely different equipment, such as boilers, furnaces, heaters, etc^1^. Almost all these pieces of equipment use fossil fuels as a source of energy. The limitations and increasing need as well as the use of these resources have led to a rise in prices and the occurrence of many environmental problems. Hence, in many countries, numerous types of research are conducted to improve the efficiency of furnaces and to reduce fuel consumption and environmental pollution^2–4^. In olefin units in petrochemical complexes, furnaces are used as the main core for generating heat for the thermal cracking of hydrocarbons and the production of unsaturated compounds^5^. One of the main problems in cracking furnaces is the production of coke inside the coils. The coke reduces the period during which the furnace is in operation and normal services and also causes environmental problems after evacuating them from the coil in the De-Coking process^6–8^. To avoid environmental problems, a solution is to return the output flow from the coil during the De-Coking operation to the combustion chamber and burn the produced coke in the furnace. The output flow from the coil during the De-Coking operation contains coke particles, air, and steam. Therefore, comparing and analyzing of the performance of olefin units (furnace and coils) during cracking process, common De-coke process and coke burning would be helpful in improving the efficiency of these process and controlling their environmental effects.

Optimizing the performance of the mentioned equipment in petrochemical complexes like any other type of energy conversion system can reduce energy consumption and reduce environmental pollution. Experimental^9,10^ and numerical^11,12^ studies have been carried out on various parameters to optimize and improve the performance of furnaces in cracking or De-Coke process^6–9^. In non-premixed combustion, the relative distance between the injection points of fuel and air, the percentage of excess air, situations of burners in the furnace and etc. are very important and some researchers studied the effect of these parameters on furnace performance^13–16^. For instance, simulating an industrial furnace to analyze the influence of excess air percent on the performance and efficiency of the furnaces showed that the increase of the excess air significantly reduces the overall temperature in the furnace, and the decrease of the excess air leads to the production of carbon monoxide^14^. In another research, Shen et al.^17^ investigated the CFD modeling for the injection of coal powder into the furnace and its impact on operating conditions. This model includes flow, thermal and chemical behavior related to coal powder and coke solid as fuels.

Furthermore, energy and exergy analysis has been proven a useful tool to estimate the performance of petrochemical complexes and to determine the quality and advantages of different types energy in a wide range of process. Knowing the energy level and the maximum potential work of each element/equipment in a process system is important for technicians and engineers to make a better decision for the system efficiency enhancement over the plant operation^18,19^. A simple definition of exergy is the maximum available work that can be gained from a thermodynamic system. Exergy analysis is originated from the first and second laws of thermodynamics. The exergy analysis estimates the energy loss and exergy destruction of elements of a targeted system through quantitative measurements^20^.

According to extensive literature review, a majority of the previous related research in various chemical and energy plants/processes focused on the exergy and energy analysis. For instance, Atienza-Marquez et al. conducted an exergy analysis to select the most suitable working fluid(s) and heat transfer fluid(s) in a liquefied natural gas (LNG) process^21^. Wang et al. used exergy analysis to compare the performance of a novel methane cracking process thermally coupled with chemical looping combustion and conventional methane cracking processes in which heat is supplied by combusting methane or hydrogen produced in the methane cracking process^22^. Mohamadi-Baghmolaei et al. worked on simulation, analytical, and numerical modeling approaches to evaluate exergy, energy, economic, and environmental analysis of a gas sweetening plant (GSP)^23^. Parvez et al., investigated energy and exergetic assessment of pyrolysis-derived gas, char and oil from gumwood under conventional and microwave heating based on the lab-scale experimental data (at 600, 700, and 800 °C)^24^. Midilli et al. evaluated some plasma gasifiers in terms of the exergetic sustainability^25^. Yan et al., evaluated thermodynamic performance of an industrial steam cracking furnace through conventional and advanced exergy analysis in order to assess its energy saving potential^26^. Also, some studies are used energy and exergy analysis in different process of H_2_ generation and CO_2_ capture, special olefin plant and Ethelyn process^27–30^. Hence, exergy analysis has been considered as an efficient tool to evaluate the performance of different biomass thermochemical routes and petrochemical process and reduced energy consumption, improved economic performance, and enhanced system efficiency.

Moreover, it is observed some methods have been proposed to reduce the formation of coke in the coils in a large number of literatures^31,32^, but however none of them are studied to produce zero coke production. Although, the burning the solid particles, such as coal, is very common, less attention has been paid to the burning of coke in the flow of De-Coking operation in the olefin units. Moreover, it was found that the burning of coke produced in coils of olefin furnaces, which is a particular type of coke, has not been investigated.

This research investigates the combustion of the coke particles, that are in the stream of the output of the coil in the De-Coke operation while injected into the furnace. Firstly, energy and exergy analysis is used to evaluate the performance of De-Coke process via burning coke in furnace and comparing this approach to the conventional process in olefin units. Secondly, strategies are developed to study the conditions for burning coke particles in the furnace of the olefin plant which are analyzed numerically using the CFD techniques. The impact of increasing De-Coke stream temperature on the coke conversion is investigated, examining the effects of varying dry air flow rates on coke particle injection. Finally, the results of these two key approaches are compared.

## Materials and methods

### Description of furnaces, burners and De-Coke process

**Fig. 1:**
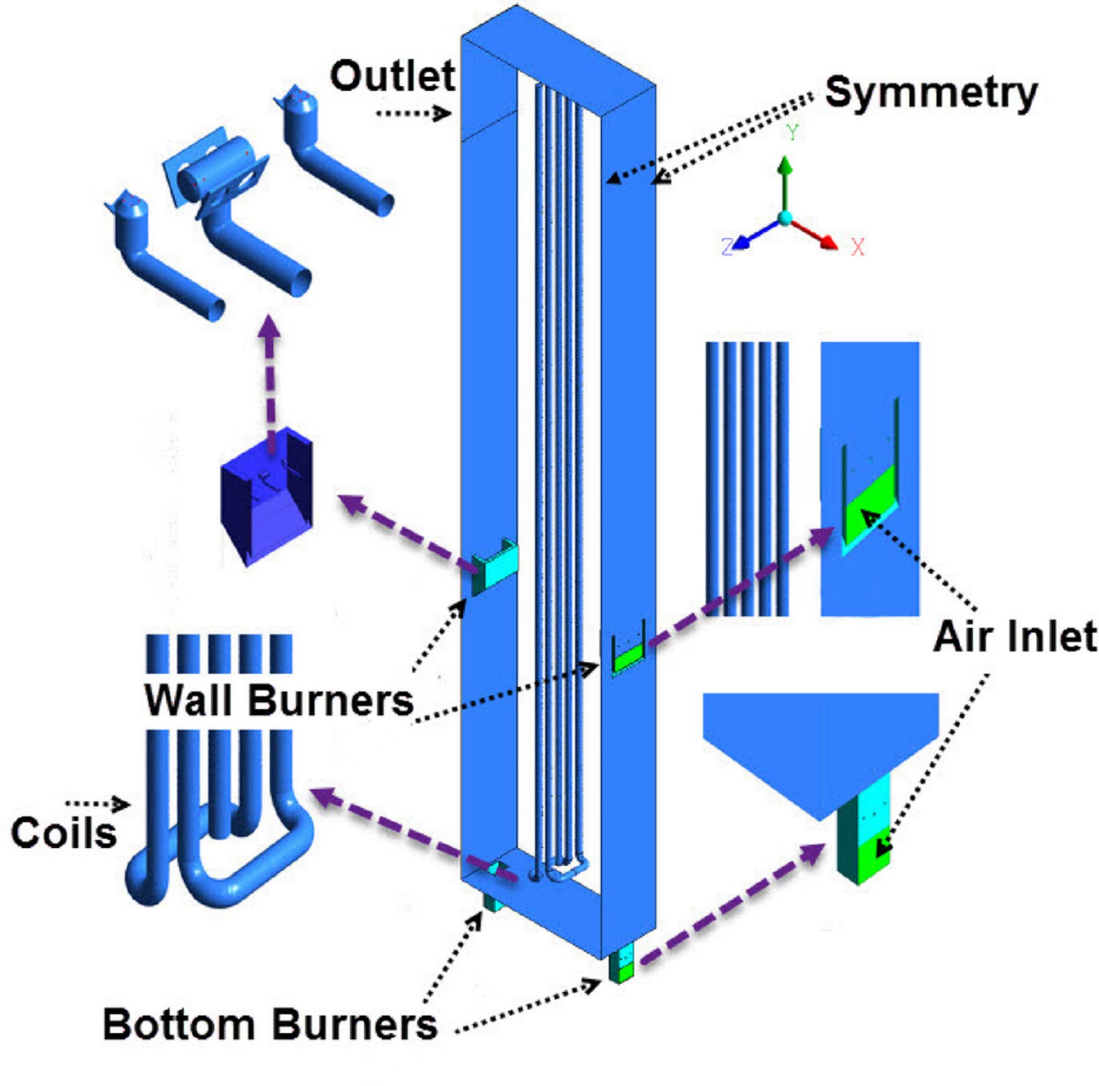
Different part of the modeled furnace.

The furnace is room furnace type with burners on both sides. Firebox dimensions are 14.66 m in height, 20.16 m in length, and 2.8 m in width, which is divided into six zones in the length direction, and each zone was divided into three identical parts. In other word, firebox including 18 identical parts in the length direction containing 90 coil passes, 36 wall burners, and 36 bottom burners. Each part of the firebox containing 5 coil passes, two wall burners and two bottom burners. Due to the presence of periodic boundary condition in each part, only one part of the furnace is modeled. A schematic overview of the simulated furnace was shown in Fig. 1.

The furnace consists of two rows of burner boxes in the floor and wall, which cause to make the heat flux uniformly distributed in the furnace. The coils in the firebox are located vertically in the middle of it and receive radiation temperature from the bottom and wall burners on both sides. Each box on the floor and wall contains several burners, that Fig. 1 illustrates an image of them. In this furnace, combustion air is supplied by a fan, which is located on top of stack, and the fan is responsible for creating a suction throughout the furnace. Specifications of the modeled furnace and burners are given in Table 1.

**Table 1:** Specification of the modeled furnace and burners.

Radiation section of the furnace (modeled part)		Box burners	
Parameters	Values	Parameters	Value
Furnace height	820 mm	Space between the floor burners	14,660 mm
Furnace length	500 mm	Space between the wall burners	1120 mm
Furnace width	20	Number of injection nozzles for each box	2804 mm
Output height	2 mm	Nozzle diameter	1700 mm
Number of modeled coils			5 passes
Number of coils in the furnace			90 passes
Coil diameter			110 mm

The performance of the furnace is that the flow of ethane feed enters the coils and by heat absorption, cracking occurs to produce ethylene. Due to the formation of coke and its deposition on the inner wall of the coils in the cracking conditions, after 30 to 45 days, in order to eliminate the coke deposition and their eclipses, the furnace enters the De-Coke operation by steam and air and the total amount of coke produced in the De-Coke operation is 1140 kg during the 44 h.

**Fig. 2:**
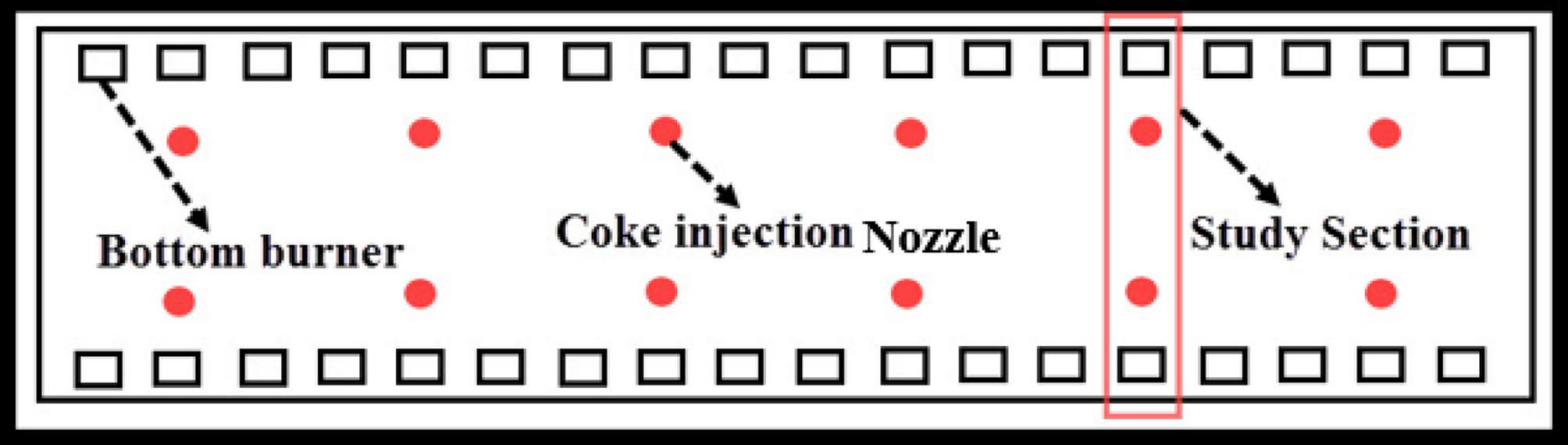
Top view of furnace bottom and Coke injection Nozzles (red circles).

**Fig. 3:**
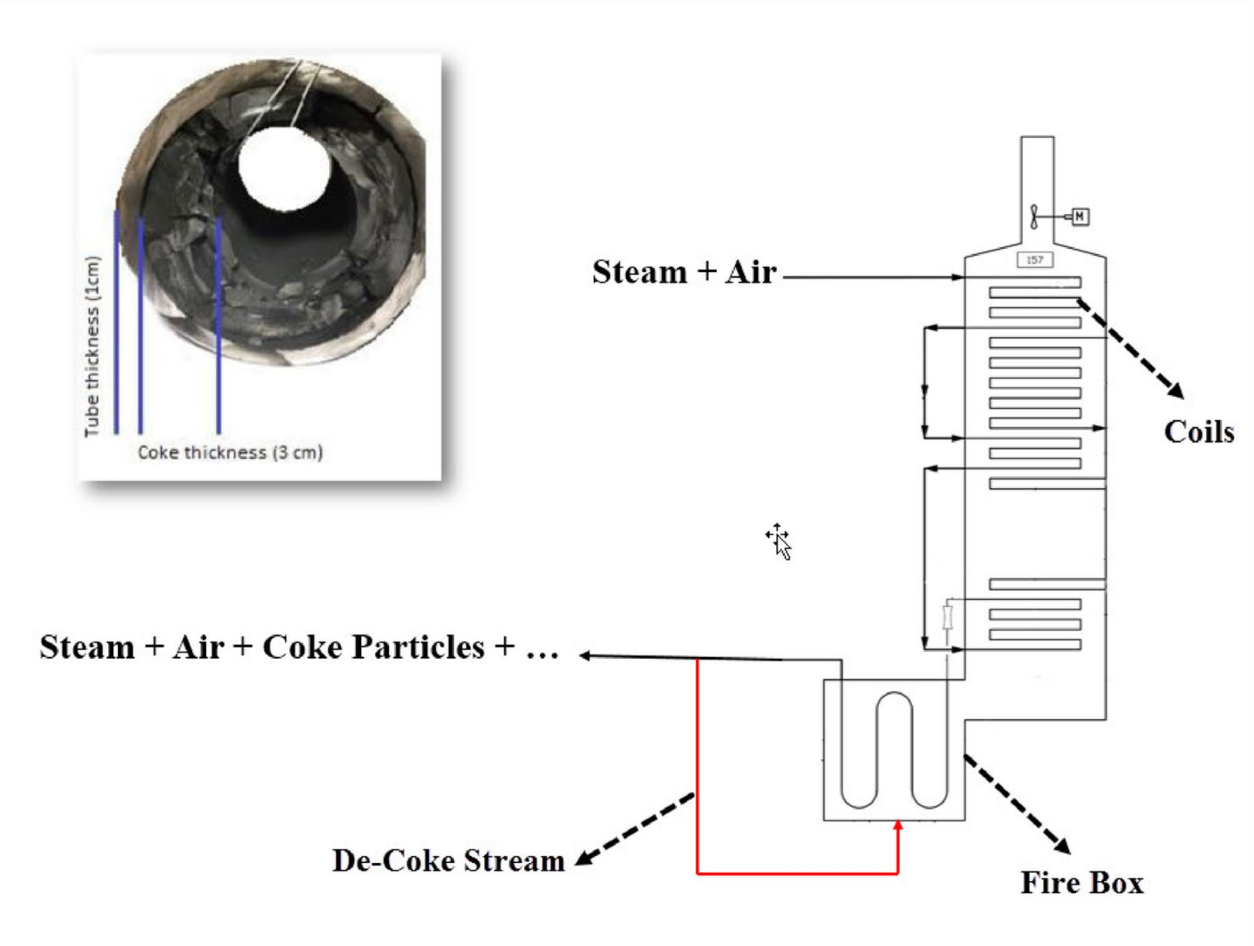
Schematic of De-Coke stream injection into firebox and coke thickness into inner surface of coils.

In this study, at first, the burning of the De-Coke stream with all components (coke particle with steam and air) was investigated by injecting through coke injection nozzles with 8 in diameter. According to Fig. 2, the simulation for furnace is conducted assuming that the flow of De-Coke is divided into 12 lines of 8 in and injected into the furnace. Injection points are considered at the furnace floor between coils and furnace walls. Figure 3 illustrated the schematic of De-Coke stream injection into firebox (red line) and coke thickness into inner surface of coils.

### Energy and exergy analysis

#### Energy analysis

**Fig. 4:**
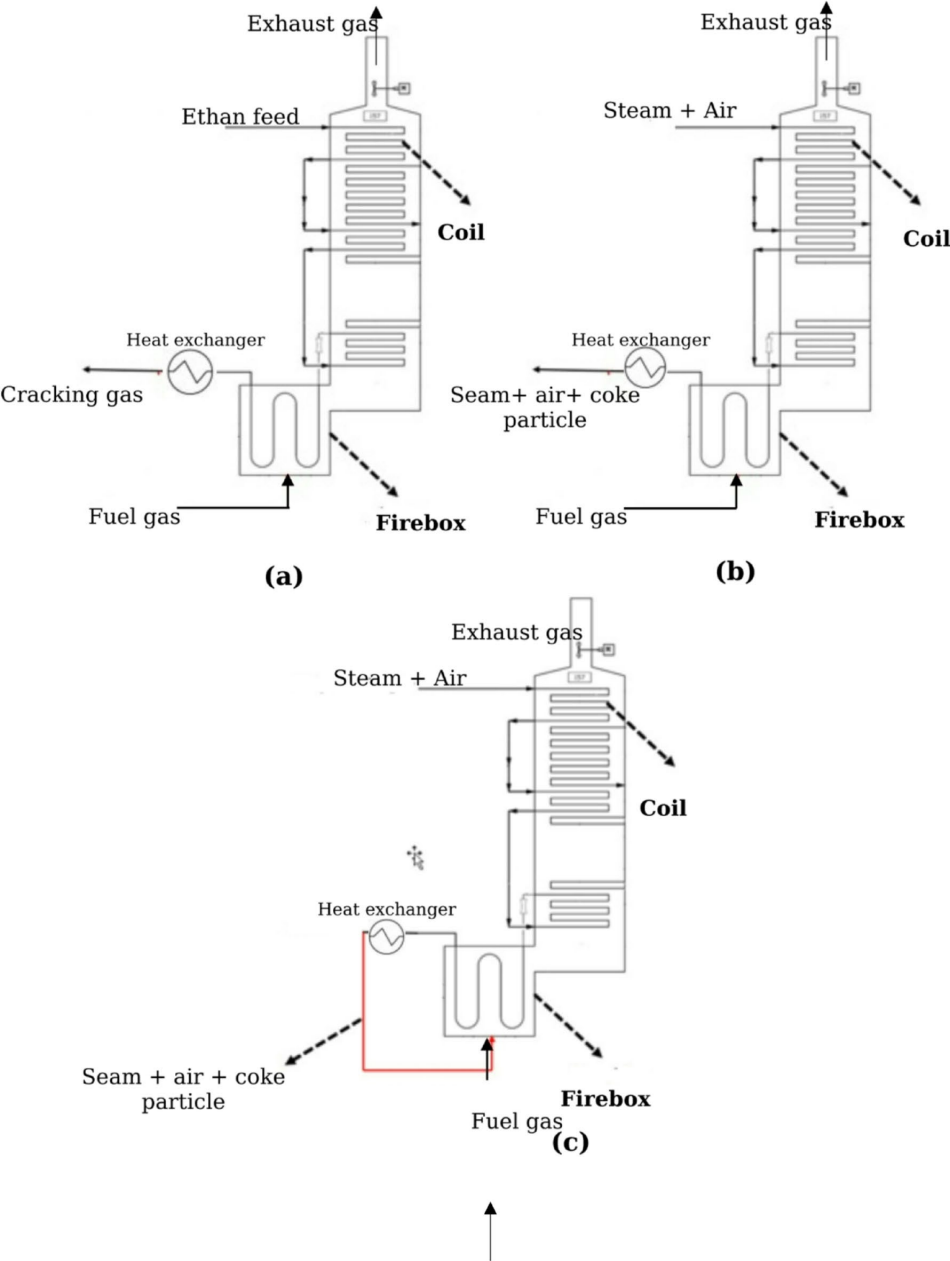
Schematic of input and output energy stream into firebox and coil for (**a**) cracking process (**b**) conventional De-Coke process (**c**) coke-burning De-Coke process.

For energy and exergy evaluation, input and output streams of each system are taken into consideration. The energy inputs are considered, i.e., the heat and the energy contained in the hydrocarbon, air, steam or other gases, whilst the outputs are the energy wasted, produced or recovered as heat transfer, gaseous product and emission exhaust. The schematic of input and output energy stream into firebox and coil for cracking process, conventional De-Coke process and coke-burning De-Coke process are shown in Fig. 4.

The cracking and De-Coke systems studied are all assumed to operate in a steady state. For a steady state system, the mass balance and energy balance equation can be expressed by Eq. (1) and Eq. (2), respectively.

1 $$\:\sum\:{\dot{m}}_{in}=\sum\:{\dot{m}}_{out}$$

2 $$\:\sum\:{\dot{E}n}_{in}=\sum\:{\dot{E}n}_{out}$$

In systems studied most often the changes in kinetic and potential energy are negligible, and mechanical works is zero for these processes, so energy balance for the aforementioned systems (Fig. 4) can be written as following^25^:

3 $$Q_{{in}} + \sum {\mathop {\dot{m}_{{in}} \left( {h_{{in}}^{0} + \Delta h_{{in}} } \right)}\limits^{ \cdot } } = \dot{Q}_{{out}} + \sum {\dot{m}_{{out}} \left( {h_{{out}}^{0} + \Delta h_{{out}} } \right)}$$

In the above equation, Q is the rate of net transferred heat, h and ∆h are formation enthalpy and enthalpy change, while *in* and *out* as indexes shows inlet and outlet flows. Generally, as can be seen for the cracking process air and fuel energy are input mass energy as well as emission exhaust of furnace as output mass energy. Moreover, there would be the heat losses as heat transfer from furnace and the heat energy that is transferred to the coils. By, taking coils in cracking process, air, steam and hydrocarbons are input mass energy and heat absorbed by the coils in the furnace would be another input energy, while the hydrocarbons gas products are the only output energy.

To comprehensively evaluate cracking and De-Coke process, energy ratio of each part or process energy efficiency of the mentioned process can be calculated based on the total energy input as Eq. (4).

4 $$\:\eta\:=\frac{{En}_{out}}{{En}_{in}}$$

#### Exergy analysis

Exergy of a substance is defined as the maximum obtainable work the substance can generate when it is brought reversibly to a state of thermodynamic equilibrium with the environment which is assumed to be at 25 C (T_0_) and 1 atm (P_0_) in this study. Exergy analysis of a process is the assessment of the conservation of mass and energy with the second law of thermodynamics. Three forms of exergy transfer are usually established to perform an exergy analysis of a system including: work interaction, heat interaction, and material streams^20^. The exergy rate balance for the aforementioned system at steady state can be written as follows:

5 $$\:{\dot{Ex}}_{in}-{\dot{Ex}}_{out}={\dot{Ex}}_{dest}$$

Where *Ex*_*in*_ and *Ex*_*out*_ denote input exergy and output energy via mass and heat according Eq. (5), *Ex*_*dest*_ shows destroyed exergy. Exergy analysis focuses on a system’s exergy flows, destruction, waste, and efficiency. For a steady-state system, the exergy destruction is the difference between the total amount of exergy into and out of the system, defined in Eq. (5). The destroyed exergy (*Ex*_*dest*_) measures the unrecoverable lost capability to do work. The maximum work can be obtained from a heat source at temperature T is governed by the Carnot efficiency, therefore the exergy of heat *Q* can be calculated by Eq. (6)^33^

6 $$\:{Ex}_{Q}=Q\left(1-\frac{{T}_{0}}{T}\right)$$

In this work, assuming negligible kinetic and potential exergy, the total exergy associated with a material stream is the sum of physical exergy (*Ex*_*ph*_) and chemical exergy (*Ex*_*ch*_). The physical exergy of a material stream can be calculated by Eq. (7)^33^.

7 $$\:{Ex}_{ph}=\sum\:{\dot{m}}_{in\:or\:out}\:\left(\left({h}_{in\:or\:out}-{h}_{0}\right)-{T}_{0}\left({s}_{in\:or\:out}-{s}_{0}\right)\right)\:$$

where *h* and *s* denote the specific enthalpy and entropy of the material stream at actual conditions (*T*, *p*), *h*_*0*_ and *s*_*0*_ denote the specific enthalpy and entropy of the material stream at environmental conditions. All enthalpy and entropy values in the above equation can be read from thermodynamics Tables and the equations describing the specific heat of the exhaust gas components^34,35^. The chemical exergy of a gas mixture can be calculated with Eq. (8)^22^.

8 $$\:{Ex}_{ch}=\sum\:{x}_{i}{Ex}_{ch,i}^{0}+R{T}_{0}\sum\:{x}_{i}ln{x}_{i}$$

Where *R* denotes the gas constant; *x*_*i*_ denotes the molar fraction of component *i* in the mixture, and *Ex*^*0*^_*chi*_ denotes the standard chemical molar exergy of component *i*. The detailed calculation method of the exergy of work, heat and material streams can also be found in some other studies^33,36,37^. Also, the mole fraction of materials used in this work is listed in Table 2.

Finally, the exergy efficiency of these cracking and De-Coke processes is defined as the ratio of exergy recovered in products or heat to the total exergy delivered into the system and can be calculated as Eq. (9).

9 $$\:\eta\:=\frac{{Ex}_{out}}{{Ex}_{in}}$$

### Numerical simulation

#### CFD modeling

**Fig. 5:**
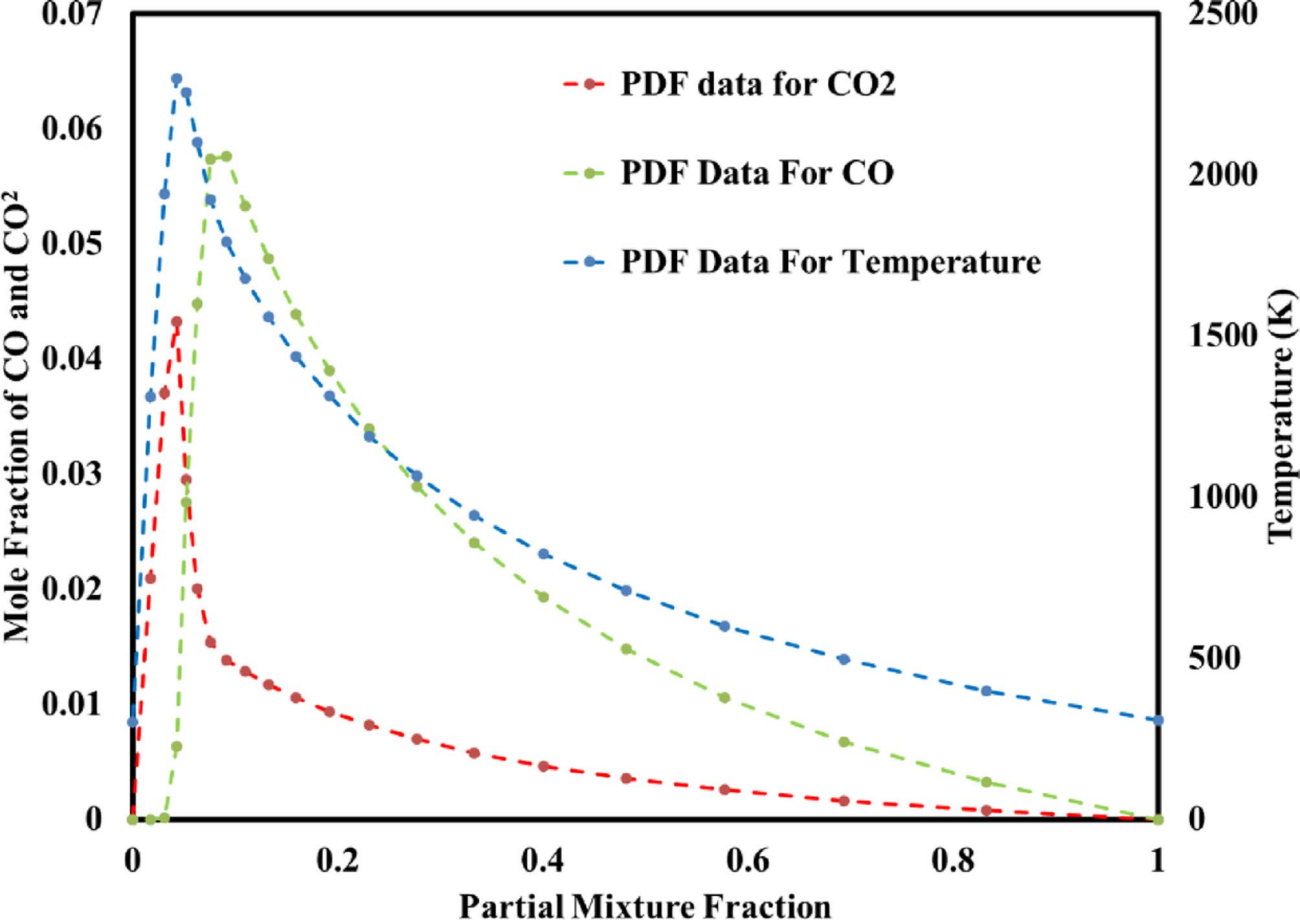
Partial fraction vs. mean temperature, CO_2_ mole fraction and CO mole fraction for PDF model.

CFD modeling includes the numerical solution of conservation equations, which in ANSYS Fluent commercial software has been used^38^. The applied models in this simulation were Realizable k-ε to model the flow of combustion gases^39^, Discrete Ordinate (DO) to model the radiation of combustion^40^, Weighted Sum of Gray-Gas Model to calculate the absorption coefficient, Euler-Lagrange approach for modelling of the motion of coke particles and the interaction between both phases of the fluid and the particle phase^41^, Intrinsic kinetic model for modeling of kinetics of coke burning^42^, Non-premixed method for modelling combustion^43^ and Probability Density Function (PDF) transport equation, had been supposed to develop the model for combustion simulation, fluid and particle flow as well as heat and mass transfer inside the furnace^44^. Figure 5 was used by PDF transport equation to calculate various quantities of temperature, CO_2_ mole fraction and CO mole fraction inside the furnace, respectively.

Moreover, the continuous phase equations in the Euler-Lagrange approach include continuity, momentum conservation, and energy conservation equations, respectively, which are as follows:

10 $$\frac{{\partial {\rho _f}}}{{\partial t}}+\nabla \cdot ({\rho _f}{{\varvec{v}}_f})=0$$

11 $$\frac{{\partial {\rho _f}{{\varvec{v}}_f}}}{{\partial t}}+\nabla \cdot ({\rho _f}{{\varvec{v}}_f}{{\varvec{v}}_f})= - \nabla P+\nabla \cdot \tau - {{\varvec{S}}_m}+{\rho _f}{\varvec{g}}$$

12 $${\rho _f}c\left[ {\frac{{\partial T}}{{\partial t}}+{{\varvec{v}}_f}.\nabla T} \right]=\nabla .(k\nabla T)+{S_e}$$

In Eqs. (10) and (12) *ν*, *t*, *P*, *g*, *c*, *k*, *T* and *τ* are defined as velocity, time, pressure, gravity acceleration, specific heat capacity, thermal conductivity, fluid temperature and stress tensor, respectively. Moreover, *S*_*m*_ is defined as source term in motion equation and *S*_*e*_ is defined as source term in energy equation and the amount of both for single phase problems are always zero. Subscript *f* in the Eqs. 10–12 denotes the fluid phase.

13 $$\tau ={\mu _f}\left[ {\nabla {{\varvec{v}}_f}+\nabla {\varvec{v}}_{f}^{T}} \right] - \frac{2}{3}{\mu _f}\nabla .{{\varvec{v}}_f}{\varvec{I}}$$

In the discreate phase modeling by Euler-Lagrange approach, Eqs. 14–17 can be hired to calculate the changes in the momentum of the passing particles (solid phase) through the control volume, that defined by ${S_m}$. Solid particles are the discrete phase in the continuous fluid phase and they are in equilibrium with the fluid phase.

14 $${{\varvec{S}}_m}=\sum\limits_{{{n_p}}} {\frac{{{m_p}}}{{\delta V}}} {\varvec{F}}$$

In Eq. (14), *F*, ${m_p}$, $\delta V$, and ${n_p}$ are the force acting on particles, mass of particles, cell volume, and number of particles, respectively. In Eq. (15), ${F_D}$ and ${F_G}$ are drag force and gravity force, respectively. And also, Eqs. (16) and (17) illustrate them.

15 $${\varvec{F}}={{\varvec{F}}_D}+{{\varvec{F}}_G}$$

16 $$\user2{F}_{D} = 6\pi \mu _{f} r_{i} (\user2{u}_{f} - \user2{u}_{p} )$$

17 $${{\varvec{F}}_G}=\frac{{{\varvec{g}}({\rho _{_{p}}} - {\rho _{_{f}}})}}{{{\rho _{_{p}}}}}$$

In Eq. (16), *i* and *µ*_*f*_ are defined as unit vector and dynamic viscosity of fluid phase, respectively. Subscript *p* denotes for the particles. The k-ε Realizable turbulence model was used to model the motion of the combustion gases in the furnace.

18 $$\frac{\partial }{{\partial t}}(\rho k) + \frac{\partial }{{\partial x_{j} }}(\rho ku_{j} ) = \frac{\partial }{{\partial x_{j} }}\left[ {\left( {\mu + \frac{{\mu _{t} }}{{\sigma _{k} }}} \right)\frac{{\partial k}}{{\partial x_{j} }}} \right] + G_{k} + G_{b} - \rho \varepsilon - Y_{M} + S_{k}$$

19 $$\frac{\partial }{{\partial t}}(\rho \varepsilon )+\frac{\partial }{{\partial {x_j}}}(\rho \varepsilon {u_j})=\frac{\partial }{{\partial {x_j}}}\left[ {(\mu +\frac{{{\mu _t}}}{{{\sigma _\varepsilon }}})\frac{{\partial \varepsilon }}{{\partial {x_j}}}} \right]+\rho {C_1}S\varepsilon - \rho {C_2}\frac{{{\varepsilon ^2}}}{{k+\sqrt {v\varepsilon } }}+{C_{1\varepsilon }}\frac{\varepsilon }{k}{C_{3\varepsilon }}{G_b}+{S_\varepsilon }{\text{ }}\,\,\,{\text{ (19)}}$$

In Eq. (19), *C*_*1*_ constant is defined as:

20 $${C_1}=\hbox{max} \left[ {0.43,\frac{\eta }{{\eta +5}}} \right],\eta =S\frac{k}{\varepsilon },S=\sqrt {2{S_{ij}}{S_{ij}}}$$

21 $$\frac{\partial }{{\partial t}}\left( {\rho \overline{f} } \right) + \nabla \cdot \left( {\rho \overrightarrow {\nu } \overline{f} } \right) = \nabla \cdot \left( {\frac{{\mu _{t} }}{{\sigma _{t} }}\nabla \overline{f} } \right) + S_{m} + S_{{user}}$$

22 $$f=\frac{{{Z_i} - {Z_{i,ox}}}}{{{Z_{i,fuel}} - {Z_{i,ox}}}}$$

The DO model was used for modeling of radiation heat transfer. Equation (23) illustrate the DO radiation model.

23 $$\nabla .\left( {{I_\lambda }\left( {\overrightarrow r ,\overrightarrow s } \right)\overrightarrow s } \right)+\left( {{\alpha _\lambda }+{\sigma _s}} \right){I_\lambda }\left( {\overrightarrow r ,\overrightarrow s } \right)={\alpha _\lambda }{n^2}{I_{b\lambda }}+\frac{{{\sigma _S}}}{{4\pi }}\int\limits_{0} {{I_\lambda }\left( {\overrightarrow r ,\overrightarrow {s'} } \right)\Phi \left( {\overrightarrow s ,\overrightarrow {s'} } \right)} d\Omega$$

Both of *s* and *r*,* I*,* α*, and *σ* are defined as direction of the coordinate axis, intensity of radiation, thermal diffusivity, and Stephan-Boltzmann constant, respectively.

In Table 2, in addition to the above-mentioned models, the constant parameters of the simulation such as the mass flow and composition of fuel, excess air, and coke as well as data for the coke stream, and other parameters, are given. Also, the coke specifications^40^ and information about the intrinsic model is presented in Table 3. It should be noted coke samples used in experiments were obtained from a piece of radiation coil of an ethane cracker furnace as shown in Fig. 3 where were milled in the size of 1–2 mm and Table 3 illustrated the components of this coke. Moreover, the kinetics of coke combustion in the investigated processes have been thoroughly detailed in our previous studies and are readily accessible^45^.

**Table 2:** Details of the parameter values and operating condition used in the simulation for the condition of 30% excess air.

Parameters	Values
Mass flow of fuel (kg/s)	0.0399
Mass flow of air (kg/s)	0.2881
Mass flow of De-Coke (kg/s)	3.2597
Mass flow of particles (kg/s)	0.0033
Fuel gas	
The mole fraction of CH_4_	0.2203
The mole fraction of C_2_H_6_	0.0018
The mole fraction of C_2_H_4_	0.0055
The mole fraction of H_2_	0.7723
Air	
The mole fraction of N_2_	0.79
The mole fraction of O_2_	0.21
Temperature of fuel stream (K)	308
Temperature of Air stream (K)	303
Temperature of De-Coke stream (K)	454
Particle density (kg/m^3^)	1900
Particle diameter (m)	5e – 5

**Table 3:** Specification of intrinsic kinetic model and coke.

Parameters	Values
Specification of Intrinsic kinetic model	
Mass diffusion limited rate constant	5e- − 12
Kinetic limited rate pre-exponential factor	0.2215
Kinetic limited rate activation energy (J/kgmole)	8.23e + 7
Char porosity	0.01824387
Tortuosity	1.414214
Specific internal surface area (m^2^/kg)	2490.92
Mean pore radius (m)	6.7954e − 9
Specification of coke	
Ash (ASTM D3174-11)	0.8 (% wt)
Volatile (ASTEM D3175-11)	0.5 (% wt)
Fixed C (ASTM D3172-89(02))	98.4 (% wt)
Sulfur (ASTEM E1915-09)	0.1 (% wt)

#### Grid independency and model validation

After the production of furnace geometry, the furnace has meshed with the ANSYS grid generating software. To verify the grid independency, five different grids with cell numbers from 58,000 to 2,074,000 cells were created. Table 4 shows the characteristics of the prepared meshes.

**Table 4:** Specification of the grid independency.

Grid specification	Mesh_1	Mesh_2	Mesh_3	Mesh_4
Nodes	579,849	1,265,124	1,520,603	2,072,587
Elements	2,941,753	6,820,843	8,154,053	11,248,805
Min size (m)	1.e − 003	1.e − 003	8.e − 004	8.e − 004
Proximity min size (m)	1.e − 003	1.e − 003	8.e − 004	8.e − 004
Max face size (m)	0.1550	0.1250	9.e − 002	8.e − 002
Max size (m)	0.310	0.250	0.180	0.160

**Fig. 6:**
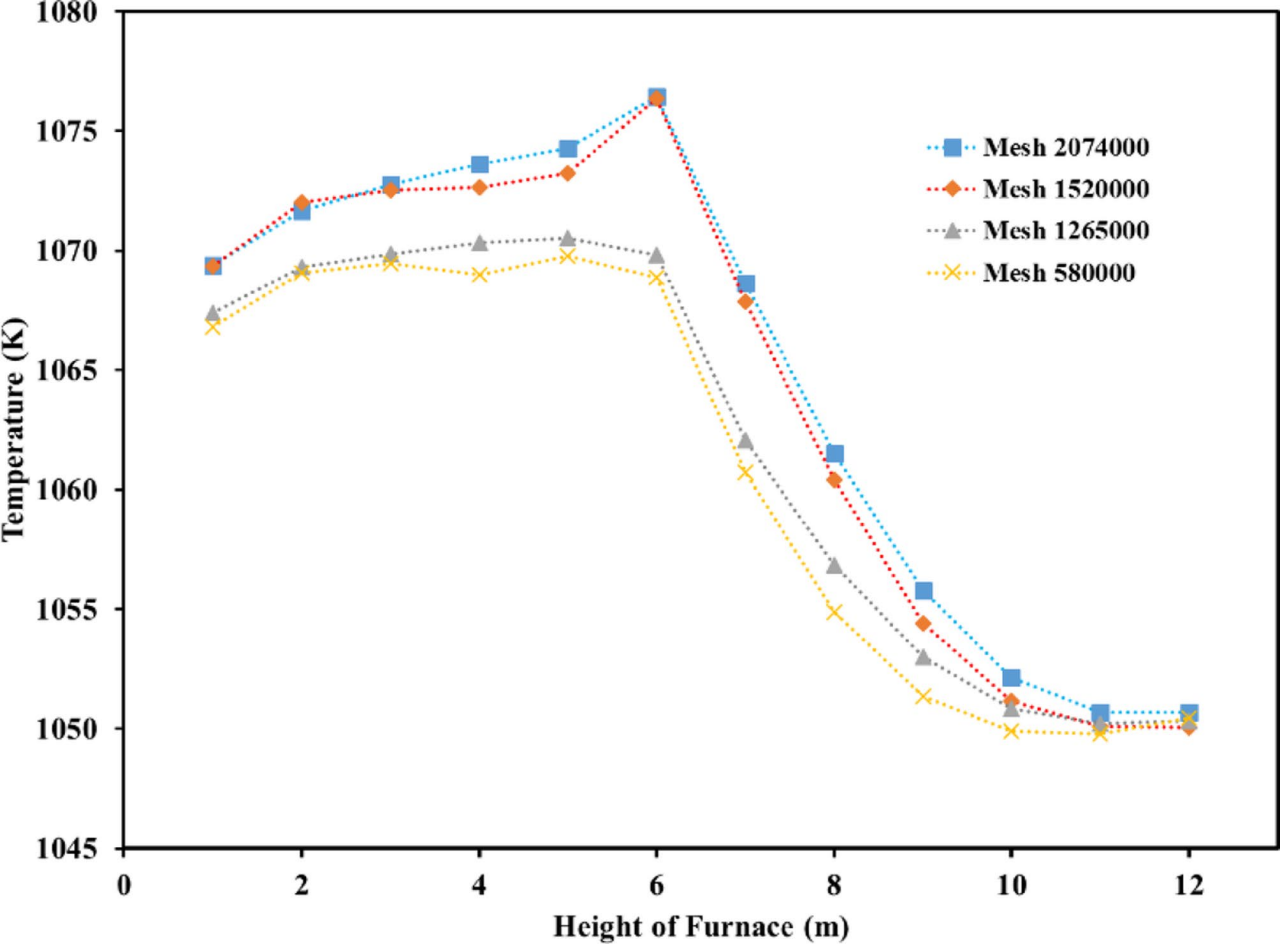
Average temperature along the furnace at difference heights obtained for four grids.

With the intention of verifying the grid independency, the average temperature and flow velocity in the different furnace heights were calculated for each of the 5 grids and the results are presented in Fig. 6. It is clear from these results that the generated grid with 1,520,000 nodes is an optimal grid because it has an equal precision to the grid with 2,074,000 nodes. This can greatly reduce the time and the cost of computing.

With the purpose of evaluating the accuracy of the simulation results, the furnace was modeled in cracking mode and without coke injection, and the results were compared with the operational data obtained from cracking furnace of the olefin unit. The output temperature and oxygen mole fraction at the furnace output are compared with the operational data and the results were shown in Table 5, with average relative error (ARE) percent. Comparison of operational and simulation data showed that there is a good agreement between operational and simulation data. The ARE (%) of the modeling results is calculated comparative to the operational data by the Eq. (10).

24 $$ARE(\% )=\frac{{\left| {{X_{CFD}} - {X_{Exp}}} \right|}}{{{X_{Exp}}}} \times 100$$

**Table 5:** Comparison between CFD results and operational data.

Parameters	Operational	Simulation	ARE (%)
Firebox outlet temperature (K)	1005	1048.7	4.3
Firebox outlet O_2_ mole fraction	0.1363	0.1477	8.4

## Results and discussion

### Energy and exergy analysis

Energy and exergy analysis were performed by using experimental and numerical data in an Olefin Unit running on three different cases including cracking, common De-Coke process and De-Coke process via burning coke in furnace. It should be noted, for each of this process the analysis is done for furnace and coil as two main and separate parts of unit, this can help with gaining a better understanding of olefin unit performance. The input energy rates $\:\left({En}_{in}\right)$, energy transfer rate to coil $\:\left({En}_{coil}\right)$, energy rate of exhaust gas flow $\:\left({En}_{ex}\right)$, and energy rate due to heat transfer to environment for the furnace and to converter for the coil $\:\left({En}_{cv}\right)$ at three different processes are summarized in Table 6. Also, the schematic of input and output energy stream into firebox and coil for three different process are illustrated in Fig. 4.

**Table 6:** Energy distribution in the furnace and the coil (kW).

Furnace	Cracking process	Conventional De-Coke process	Burning Coke De-Coke process
$\:{En}_{in}$ (kW)	51164.41	14493.04	15711.66
$\:{En}_{coil}$ (kW)	22944.10	2171.10	2159.08
$\:{En}_{cv}$ (kW)	830.16	1622.27	5595.61
$\:{En}_{ex}$ (kW)	27390.16	10699.66	7956.98
Coil			
$\:{En}_{in}$ (kW)	339577.78	8672.31	8672.31
$\:{En}_{cv}$ (kW)	22944.10	7555.69	7555.69
$\:{En}_{ex}$ (kW)	316633.68	1116.62	1116.62

The results obtained about combustion process in furnace (firebox) indicate that the input energy rates of burning-coke De-Coke process is higher than conventional De-Coke process. Although the rate of heat transfer energy for burning-coke De-coke process was significantly higher in comparison with that of conventional De-Coke process, while it shows a remarkable smaller amount of exhaust gas energy rate. Moreover, these two processes approximately transfer the same amount of energy to coil for the De-Coke process. Taking, energy distribution in coil represent that input energy rate at cracking is not comparable with these two De-Coke processes because of injecting hydrocarbon in the cracking and only air and water vapor in the De-Coke processes. Also, exhaust gas energy rate at cracking was the highest amount among outputs, while for De-Coke process energy rate of heat transfer has the highest one. It should be noted heat energy at coil is transferred to converter and it can be recovered, also the energy distribution for two type of De-Coke process is the same because of considering the same boundaries for the coil at these two types of processes.

Exergy distribution in furnace and coil is reported in Table 7. It should be noted, exergy analysis was performed by evaluating exergies associated with the inlet fuel and air, exhaust gas flow, heat transfer and destruction or system irreversibilities. The inlet/outlet exergy rate of each of the abovementioned portions are calculated for furnace and coil on three different processes by using Eq. (5) to (10). On the basis of the results shown in Table 7, the exergy rate of heat transfer and irreversibilities in furnace at coke-burning De-coke process was higher than that of conventional De-Coke process. While the exhaust gas exergy rate of coke-burning De-coke process remarkably decrease in comparison with conventional De-Coke process. Moreover, in coil the distribution of exergy is likely the same as energy distribution. In general, it is noteworthy that the input exergy rate for cracking and De-Coke process at furnace was higher than the input energy rate due to specific exergy of hydrocarbons are higher than its heating value. However, this trend for coil only works for cracking process. It can be explained with input energy to coil for De-Coke process is not in the form of hydrocarbons.

**Fig. 7:**
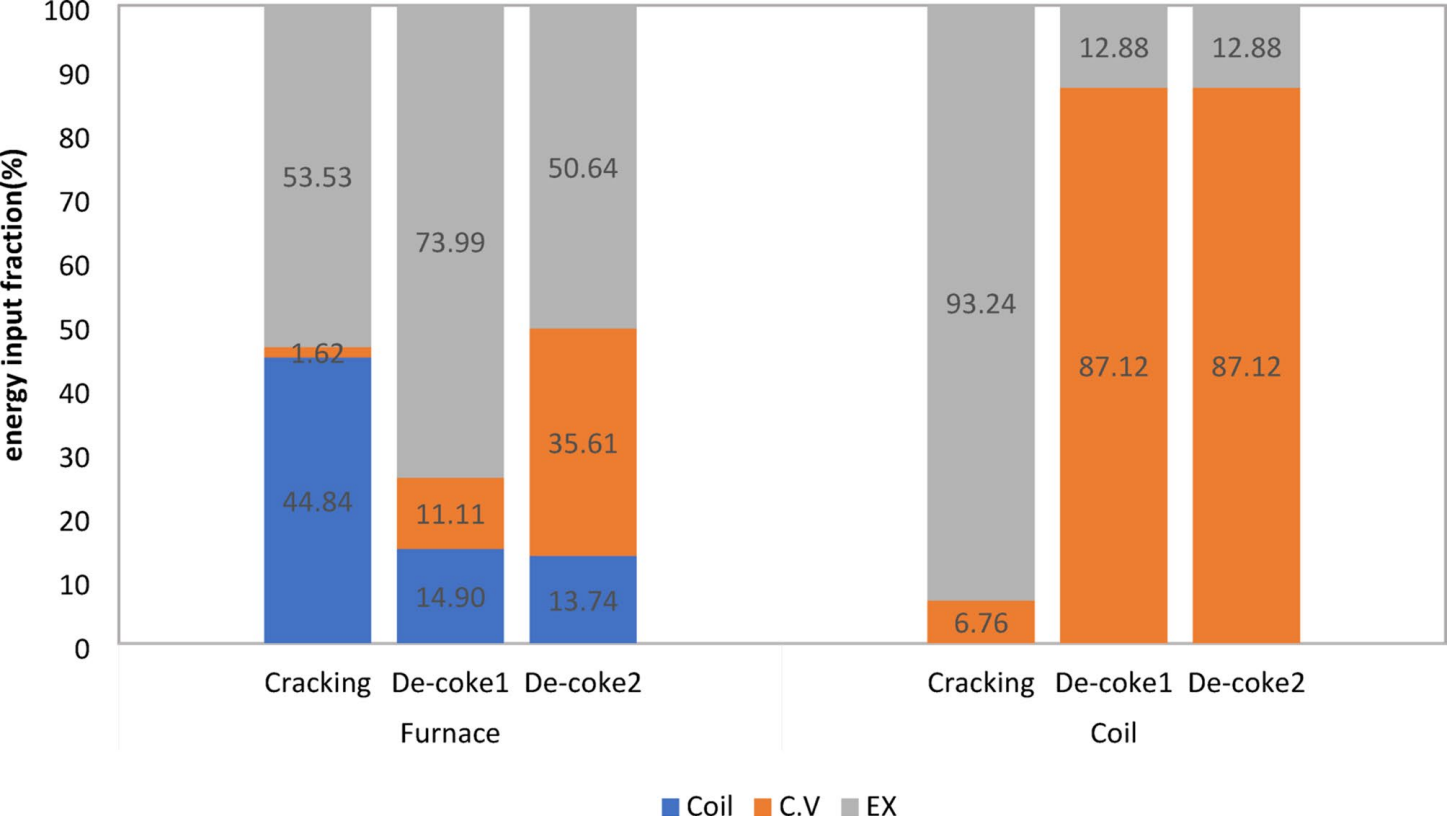
Energy fraction in furnace and coil (kW) for three process.

**Fig. 8:**
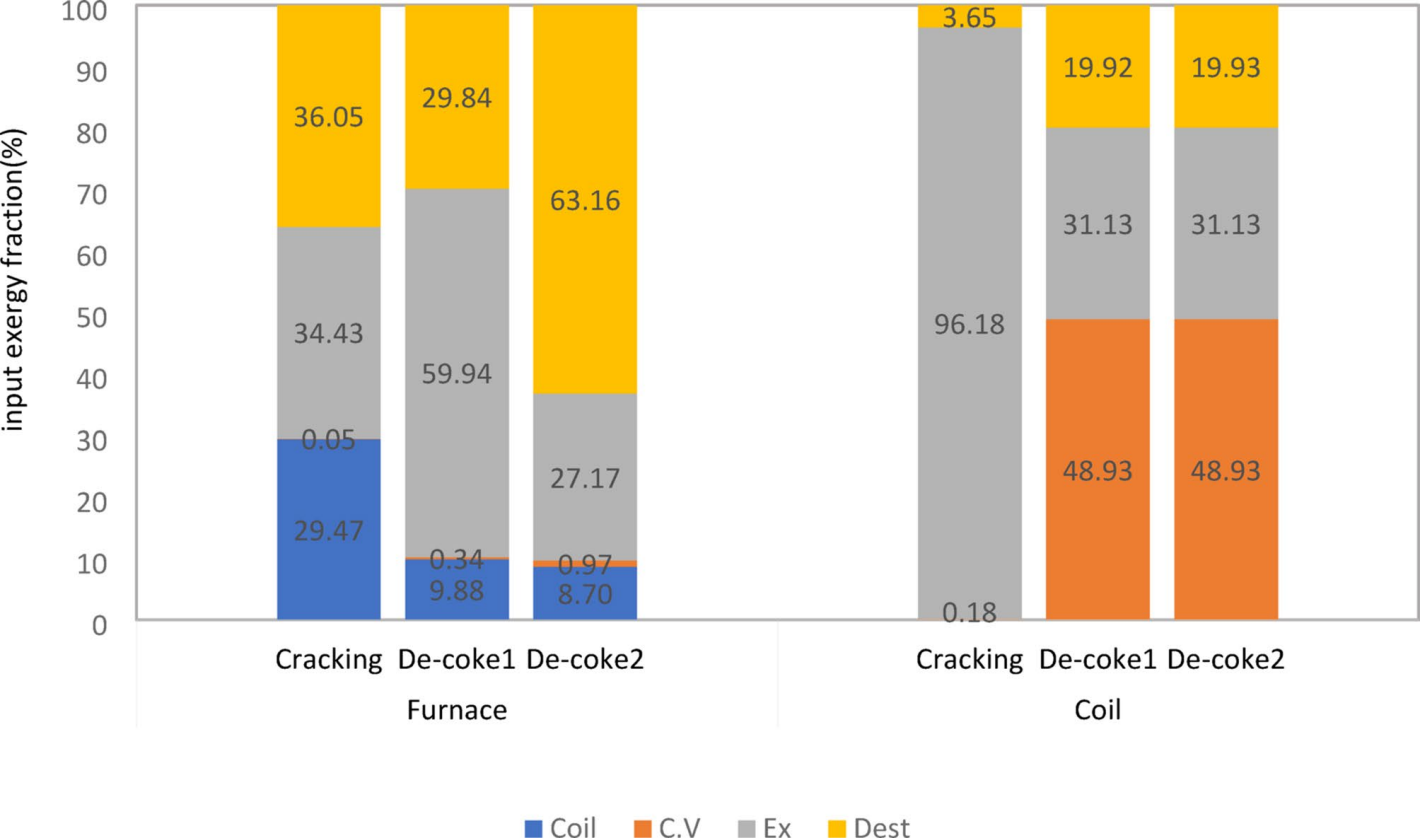
Exergy fraction in furnace and coil (kW) for three process.

In order to provide a comprehensive and deep information about energetic performance of olefin unit running on three different processes, the energy fraction of each component is calculated by dividing the individual energy quantity to the energy of the fuel plus the energy of the inlet air and other forms of energy for furnace and coil. The energy and exergy balance for furnace and coil at different process is shown in Figs. 7 and 8. It should be noted in these Fig conventional De-coke process and burning-coke process are shown with De-Coke1 and De-Coke2, respectively.

**Table 7:** Exergy distribution in the furnace and the coil (kW).

Furnace	Cracking process	Conventional De-Coke process	Burning Coke De-Coke process
$\:{Ex}_{in}$ (kW)	54496.88	15389.32	17372.97
$\:{Ex}_{coil}$ (kW)	16060.87	1519.77	1511.35
$\:{Ex}_{cv}$ (kW)	29.65	52.67	167.86
$\:{Ex}_{ex}$ (kW)	18760.83	9223.94	4720.70
$\:{Ex}_{des}$ (kW)	19648.22	4592.93	10973.04
Coil			
$\:{Ex}_{in}$ (kW)	386251.9	7024.7	7024.7
$\:{Ex}_{conv}$ (kW)	688.32	3437.83	3437.83
$\:{Ex}_{ex}$ (kW)	371482.2	2187.04	2187.04
$\:{Ex}_{dest}$ (kW)	14084.33	1399.87	1399.87

The energy balance for furnace and coil at different process is shown in Fig. 7. Taking furnace proves that the part of heat transfer to environment was less than 2% in cracking process, but at conventional and burning-coke De-Coke process it experiences a remarkable increase. Regarding exhaust gas energy shows that although the half of energy is wasted as exhaust gas for cracking and De-Coke2, this part for De-Coke1 has a higher part about 74%, therefore at furnace the maximum of energy is wasted as exhaust gas. Moreover, as can be seen roughly 45% of input energy is transferred to coil and used for cracking process at coil, while for conventional De-Coke process it was about 15%, and at De-Coke2 it was almost 7% lower than that of conventional De-Coke.

Evaluation the energy distribution at coil in Fig. 7 represent that at cracking process the main part of input energy, 93.4%, was for exhaust gas, actually it is recovered as new hydrocarbons including C_2_H_4_, C_2_H_6_ and others species as target product, and only about 7% is transferred to converter as heat. While for De-Coke processes it has a reverse trend, meaning that 87% of input energy is recovered at converter in the form of heat and 13% is wasted as exhaust gas.

A comparison of exergy distribution of furnace in Fig. 8 indicates that for three processes the lowest part of exergy was about heat transfer to environment and energy transfer to the coil, respectively. The percentage of heat transfer was negligible, however 30% of input exergy is used at coil for cracking process and this part for De-Coke process was less than 10%. Taking exhaust gas exergy indicate that although 34% of input exergy is wasted as exhaust gas in cracking process and it experience a significant increase at conventional De-Coke process, burning coke at De-Coke2 process can decrease this part of exergy significantly, it was 54% lower than that of De-Coke1 process. Moreover, the lowest level of destroyed exergy was for conventional De-Coke process, while at De-Coke 2 process is more than 60%.

Regarding exergy distribution in coil represent although the part of heat transfer exergy at cracking process was less than 0.5%, at De-Coke process roughly 50% of input exergy is transferred to converter as heat transfer. Moreover, 96% of exergy is recovered as exhaust gas in cracking process (this part can be called the utilizable exergy), and the exergy wasted as exhaust gas in De-Coke process was one third of this part in cracking process. Finally, comparing exergy destruction for three process at coil shows that this part for cracking process was only about 4% while at De-Coke process is significantly higher, about five times more than that of cracking process. These findings are consistent with the result of wang et al.^22^. They reported that exergy output and exergy destroyed of a methane cracking process for hydrogen production were about 90% and lower than 10%.

### Energy-exergy comparison

**Fig. 9:**
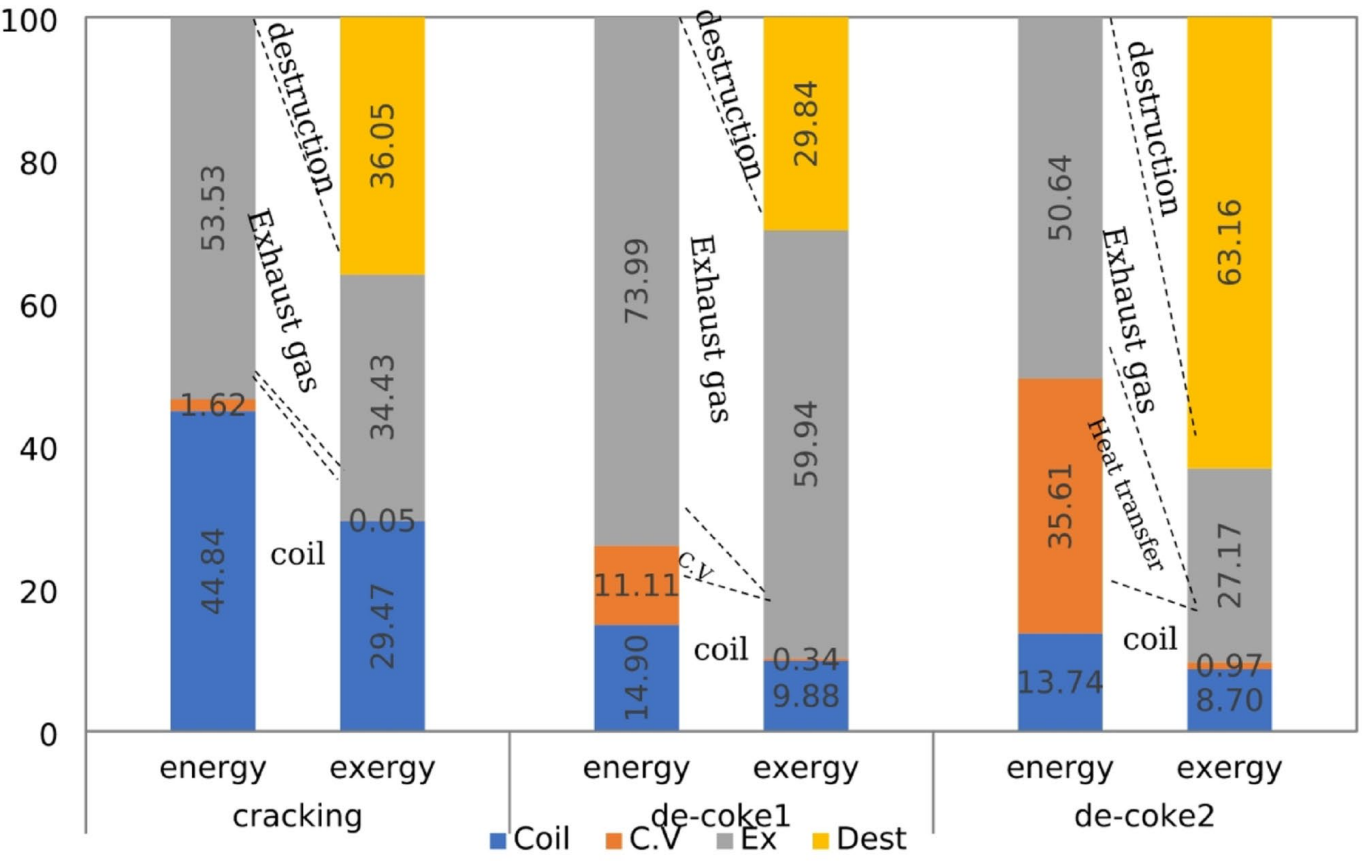
Energy -Exergy comparison in furnace (kW) for the three processes.

**Fig. 10:**
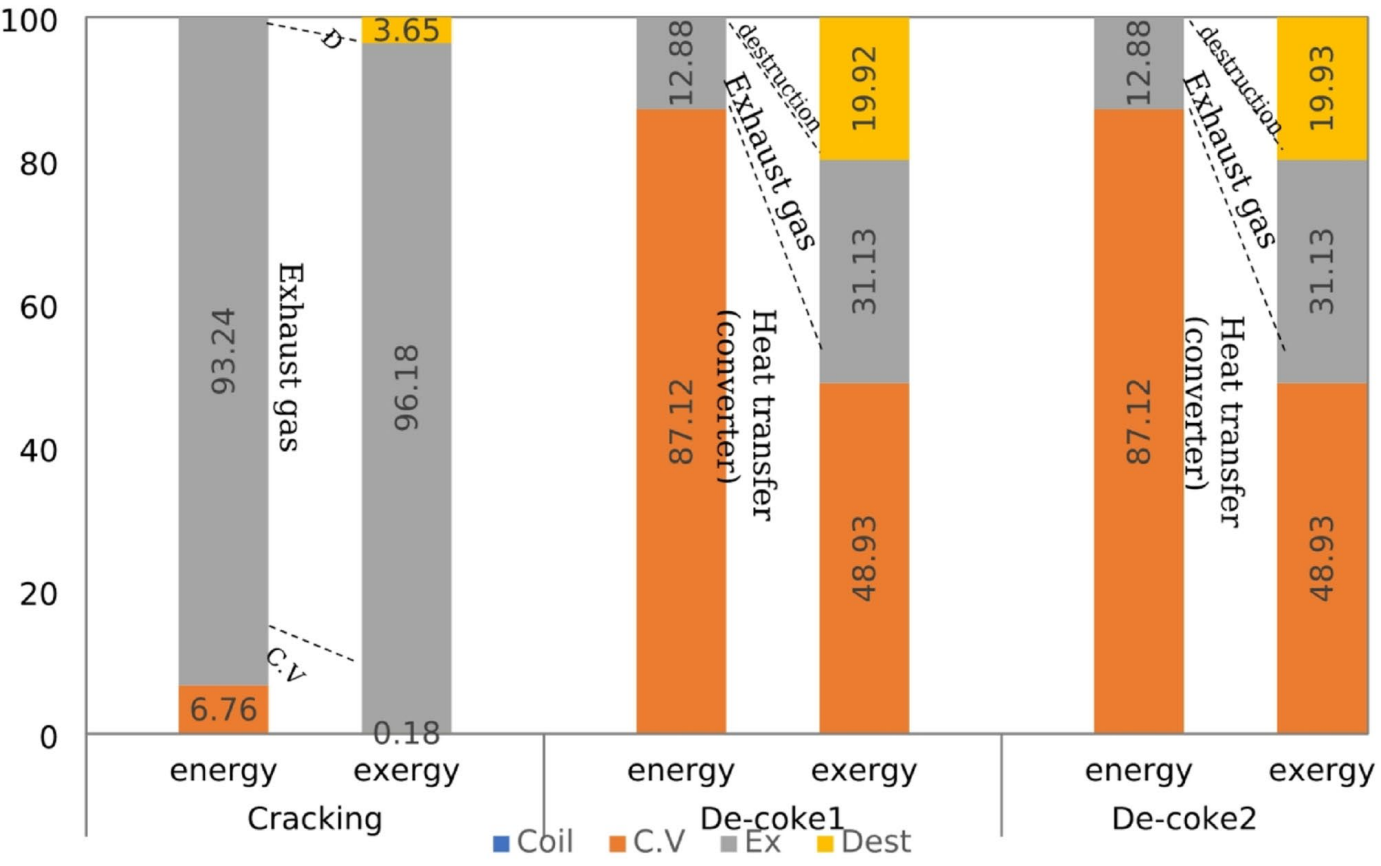
Energy–Exergy comparison in coil (kW) for three process.

The percentages of various energy and exergy terms were compared on graphs in Figs. 9 and 10 for furnace and coil at three different processes. A comparison between the part of exergy and energy which transfer to coil for three different process proves that the percentage of coil exergy (exergy efficiency) is slightly lower than the percentage of coil energy (energy efficiency), that is 34% on the average of three process. Increasing the exergy rate of fuels rather than their energy rate is the main reason for this reduction. In other words, fuel (hydrocarbon) quality or its availability is always greater than its heating value. Also, the low exergy efficiency can be due to the high exergy destruction during fuel combustion. These results are in agreement with some other studies about combustion and gasification process of biomass and hydrocarbons^25,46^.

The considerable reduction of exergy percentage for heat transfer and exhaust gas is proven through comparison with their equivalent energy percentage for different process, the reduction was about 95% for heat transfer in average. According to the definition of exergy, it can be concluded that the considerable portion of the lost energy through heat transfer are not capable of producing any work or useful energy. The percentage of this reduction for exhaust gas was lower than that of heat transfer as can be seen in Fig. 9, it was 35.68%, 18.98% and 46.34% for cracking, conventional and coke-burning De-Coke process, respectively. Actually, the percentage of exhaust gas is lower than that for heat transfer. So, the energy losses through exhaust gases have greater quality than that for heat transfer. Moreover, this comparison proves that a significant portion of energy in these systems is dissipated through irreversibility, while in the first law analysis and energy balance, losses are calculated only in the form of heat transfer and exhaust gas energy.

Comparing the exergy and energy distribution for coil at cracking, De-Coke process in Fig. 10 indicate that the percentage of exhaust gas exergy is higher than exhaust gas energy, while for heat transfer due to converter it shows a different trend, meaning that this part experiences a remarkable reduction. It can be concluded that the considerable portion of the transferred energy through converter are not capable of producing any work. Also, the energy losses through exhaust gases have greater quality than that for heat transfer. Moreover, as can be seen, about 20%of exergy in De-Coke process wasted through irreversibility, whereas for cracking process it was less than 5%. Also, generally the destroyed exergy in coil is significantly lower than the process in furnace, in other words exergy destruction in cracking and De-Coke process in coil is significantly lower than combustion in furnace.

### Numerical analysis

In this section the firebox condition with the coke injection was simulated first and then the sensitivity analysis was performed to investigate the effect of De-Coke stream temperature on the operation condition profiles and coke conversion in the firebox. Finally, the coke particles were injected into the firebox with different amount of the excess air and the results are described in the following sections.

#### Firebox operation conditions with coke injection

**Fig. 11:**
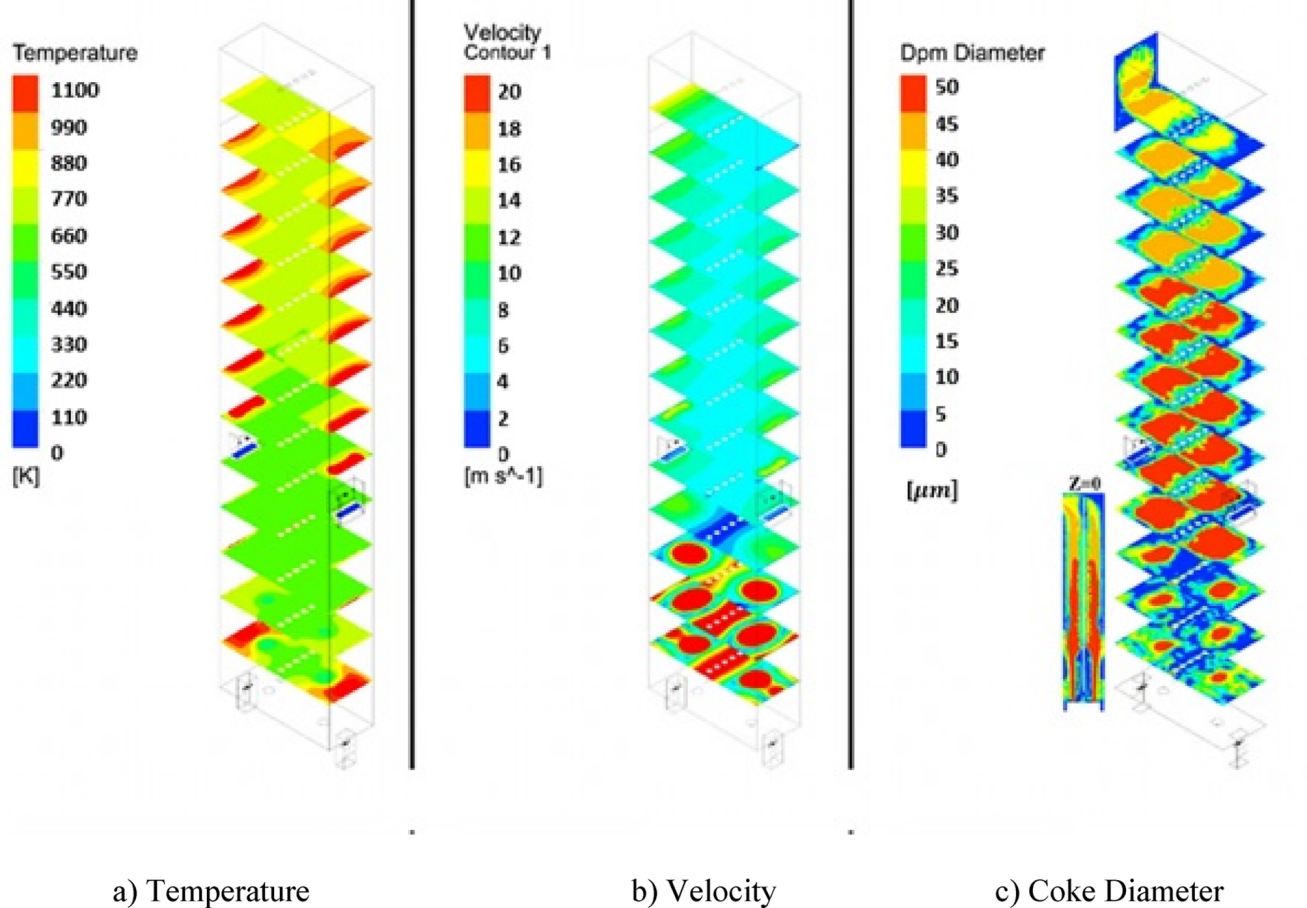
Contours for De-Coke stream injection to the furnace in operation condition.

Figure 11 shows that when the coke particles are injected into the furnace, the temperature in the entire furnace decreases and the velocity of combustion gases increases significantly in the furnace. Thus, the residence time of coke particles decrease in the furnace and the coke conversion is quite low and equals 42.36%. The reason is that the steam has a very high heat capacity and can absorb a lot of heat from the furnace. On the other hand, the flow rate of steam that entered the furnace is so high in the De-Coke operation. And according to Fig. 11, a large amount of un-reacted coke is going out from the furnace. Therefore, there is no possibility of coke burning in the furnace with the operation conditions. Therefore, it is possible to change the above-mentioned conditions to achieve better results for coke burning. These conditions are discussed in the following sections.

#### Effect of De-Coke stream temperature on the coke conversion

**Fig. 12:**
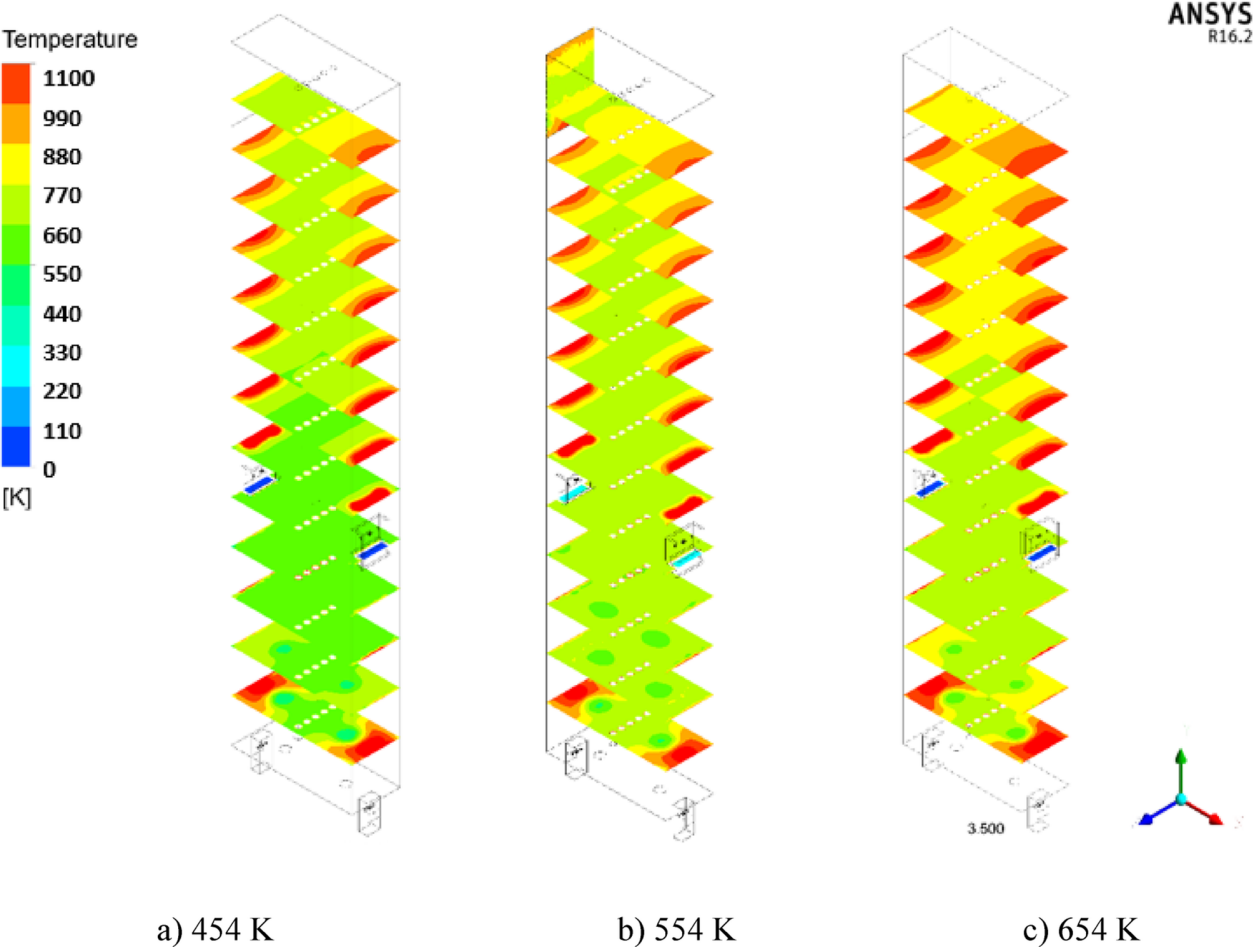
The effect of increasing De-Coke flow temperature on the furnace temperature distribution.

**Fig. 13:**
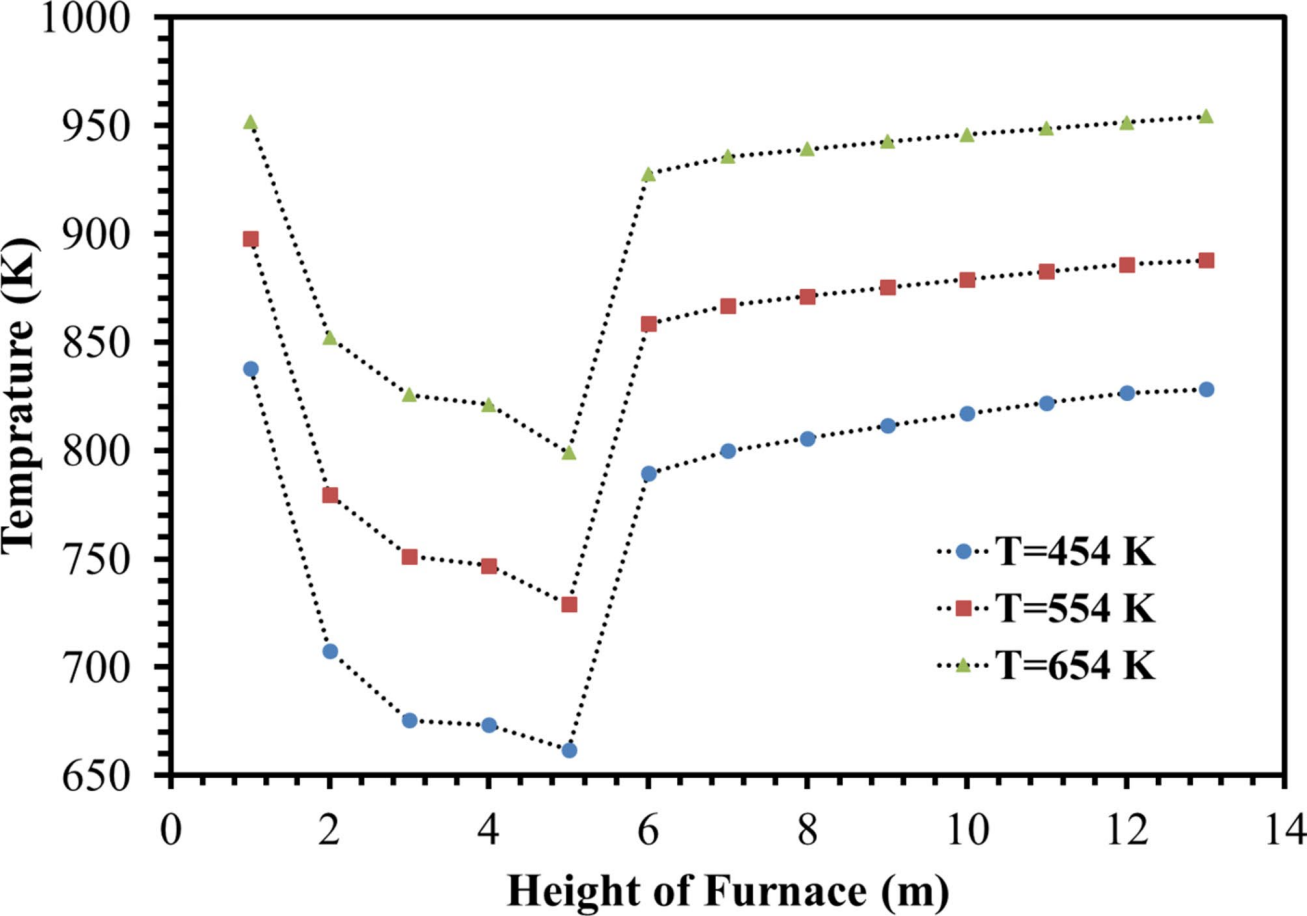
Temperature distribution in the different heights for different De-Coke flow temperature.

Three different temperatures of 454, 554 and 654 K were compared with each other to investigate the effect of increasing the flow De-Coke temperature on coke burning. From Figs. 12 and 13, which show the effect of the De-Coke stream temperature, it can be concluded that by increasing the De-Coke stream temperature, the temperature rises throughout the furnace, so the average temperature in the throughout furnace for temperatures of De-Coke stream (454, 554 and 654 K) was calculated 773.5 K, 839.3 K, and 907 K respectively. The results showed that with increasing temperature of De-Coke stream from 454 K to 554 K and from 554 K to 654 K, respectively, its conversion can be increased by 7.84% and 14.75%, respectively. As can be seen from Fig. 13, the inlet temperature is higher, which leads to a higher temperature in the Firebox space. Also, the reason for the sudden decrease in temperature at the height of about 5 m of the furnace is burners on the wall of the furnace at this height. The rise in temperature is a positive factor in increasing the coke conversion. It should be noted they represent face-averaged temperature at selected surface locations.

**Fig. 14:**
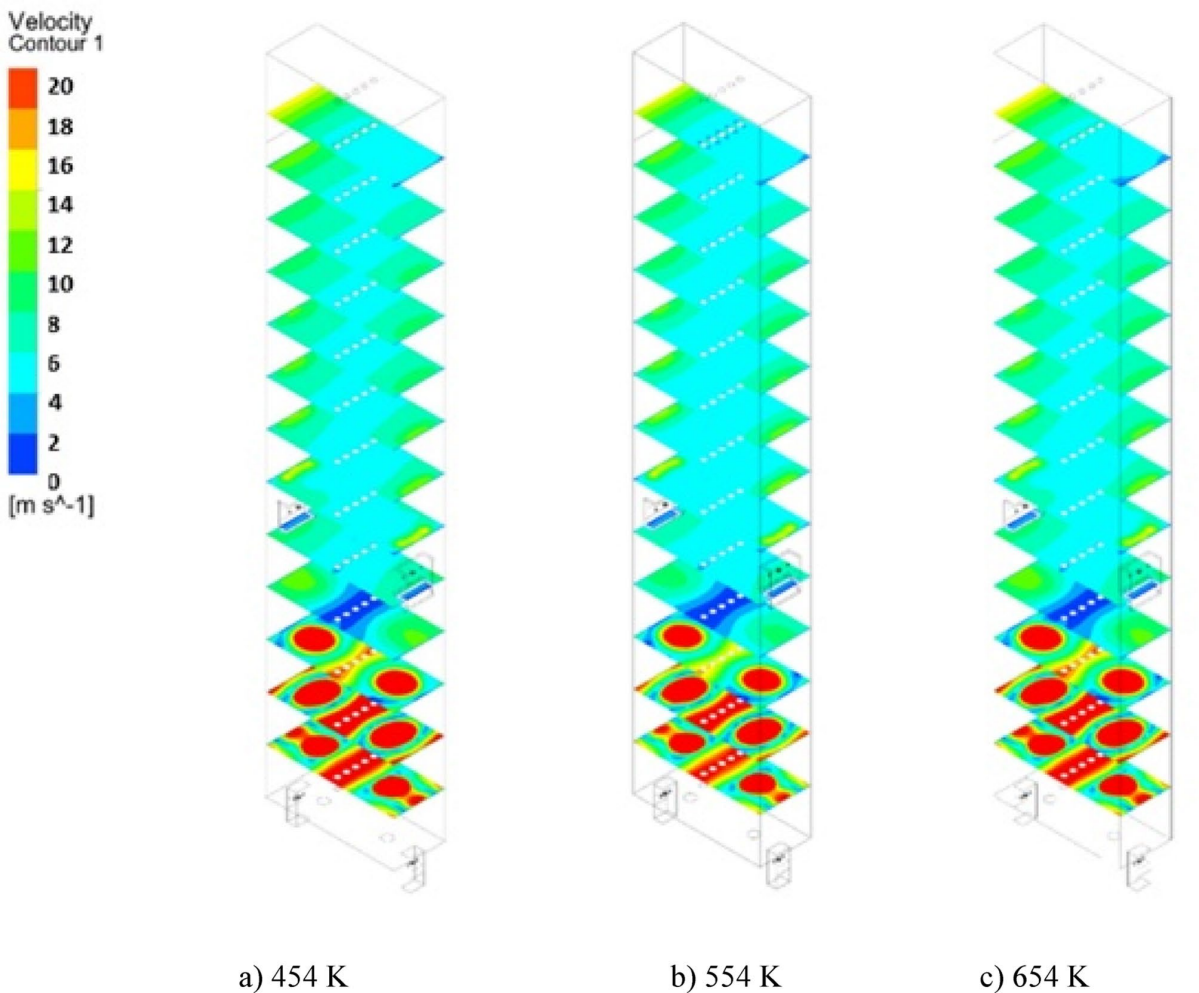
The effect of increasing De-Coke stream temperature on the velocity distribution in the furnace.

**Fig. 15:**
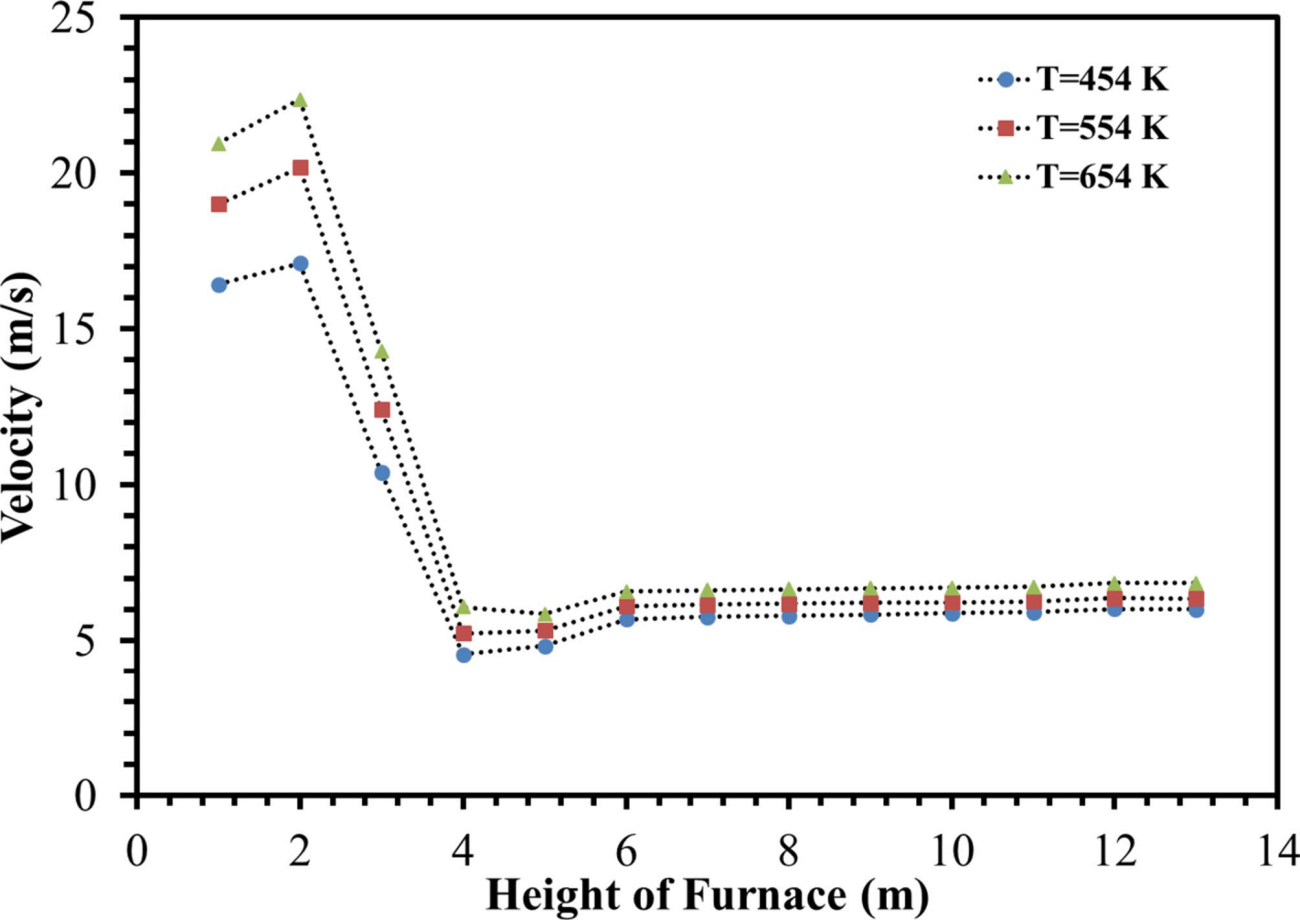
Velocity distribution in difference heights of the furnace in different De-Coke stream temperatures.

Figures 14 and 15 illustrate the velocity distribution contours and chart of the velocity at Iso-Surfaces which there is the one-meter distance between them, along with the y-axis. These results indicate, by increasing temperature of De-Coke stream from 454 K to 554 K, the velocity of combustion gases inside the furnace rises from 7.7 m/s to 8.6 m/s, and also with increasing the temperature of De-Coke stream from 554 K to 654 K, the velocity of combustion gases inside the furnace progresses from 8.6 m/s to 9.4 m/s, so the velocity of combustion gases inside the furnace increases10.5% and 18.6% for the 554 K and 654 K respectively. Increasing the velocity of gases inside the furnace reduces the residence time of coke particles, which is a negative factor. As can be seen in the Fig. 15, at a height of about 4 m from the furnace floor, the flow is developed, and the flow velocity, the averaged one at selected surface location, remains almost constant. In addition, the higher temperature of the furnace leads to higher combustion of coke particles, and more gas products are produced, which itself leads to a relative increase in the flow rate.

**Fig. 16:**
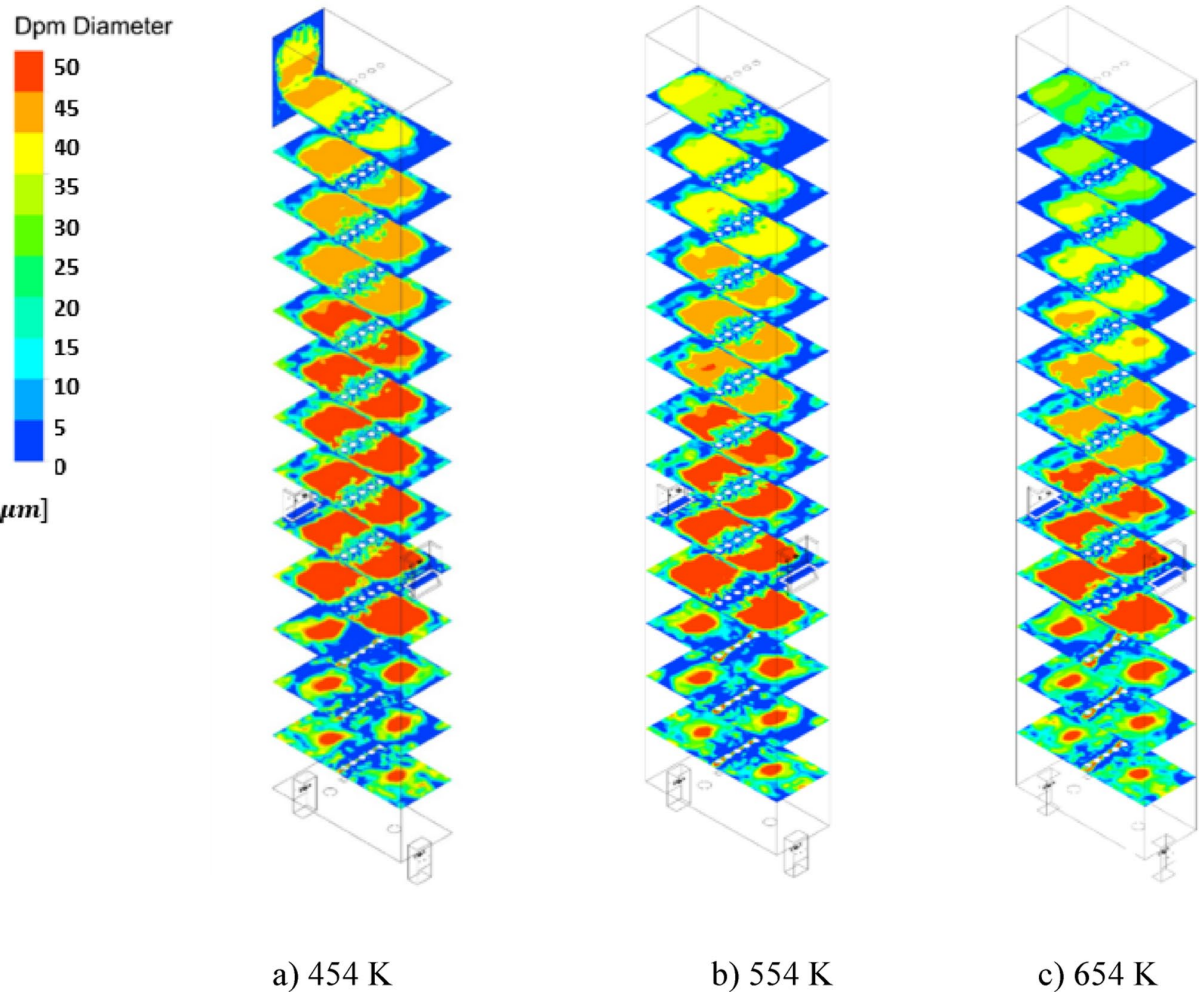
The effect of increasing De-Coke stream temperature on the particle size distribution in the furnace.

**Fig. 17:**
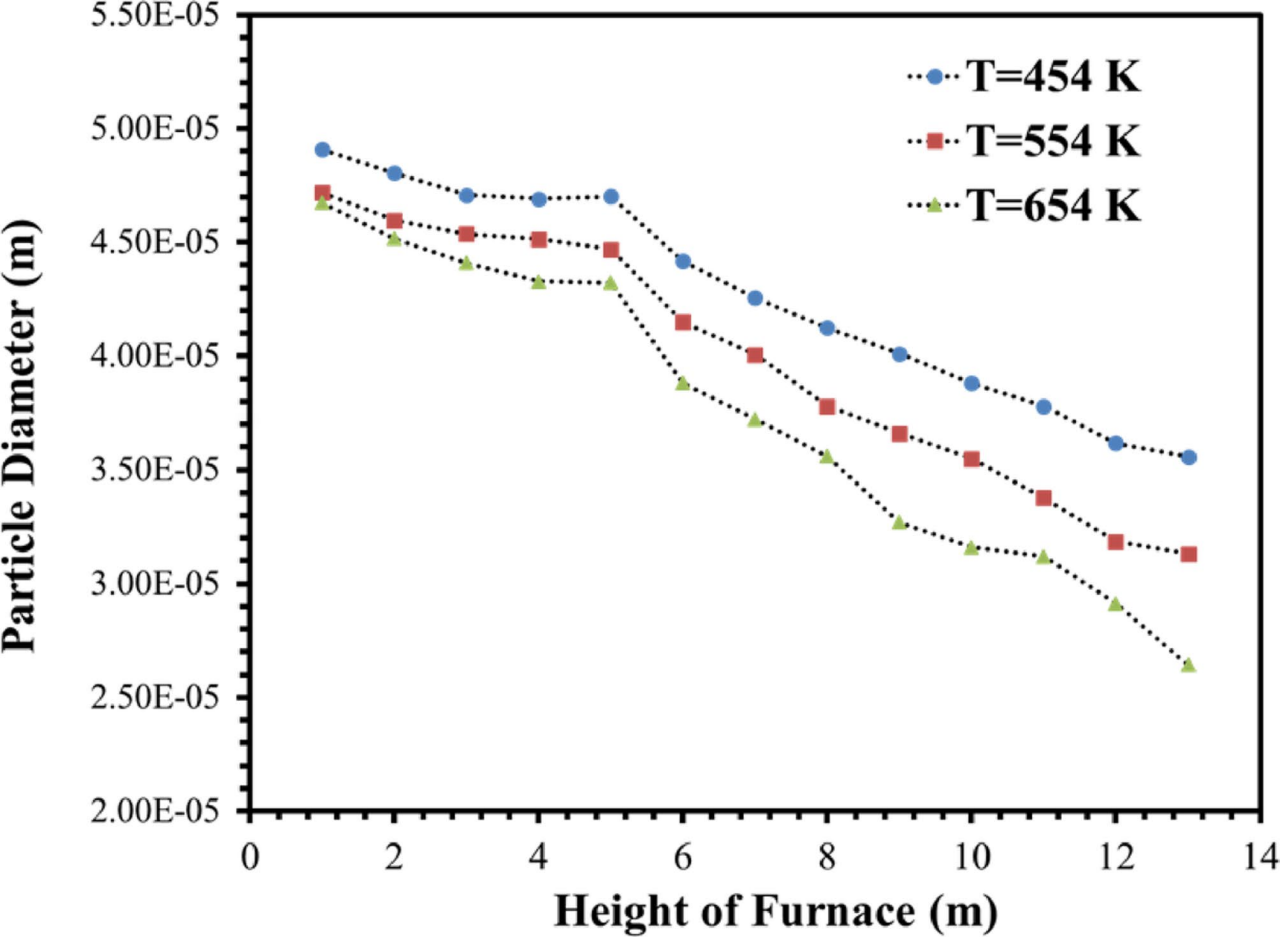
The faced-averaged particle size distribution in the different heights of furnaces at the various De-Coke stream temperatures.

Figures 16 and 17 demonstrate the coke diameter distribution contours and chart of the coke diameter at Iso-Surfaces which there is a one-meter distance between them, along with the y-axis as mentioned. These results show, by increasing the temperature of the De-Coke stream the diameter of the coke particles in the furnace decreases. As seen from Figs. 16 and 17, by increasing the temperature of De-Coke stream *the average diameter of the particles* at 454 K over the whole furnace was 4.14e-5 m, at 554 K, 3.84e-5 m and in the state of 654 K, 3.55e-5 m, so the diameter of coke particles inside the furnace decreases 7.17% and 4.53% for the 554 K and 654 K compared with 454 K, respectively. In the explanation, it can be said that higher temperature leads to more conversion of coked particles, as a result, the size of coke particles decreases. Furthermore, in the furnace, with the increase in the height of the furnace, due to the increase in the conversion of coke particles, the size of the particles decreases.

#### Coke particle injection with ambient air

**Fig. 18:**
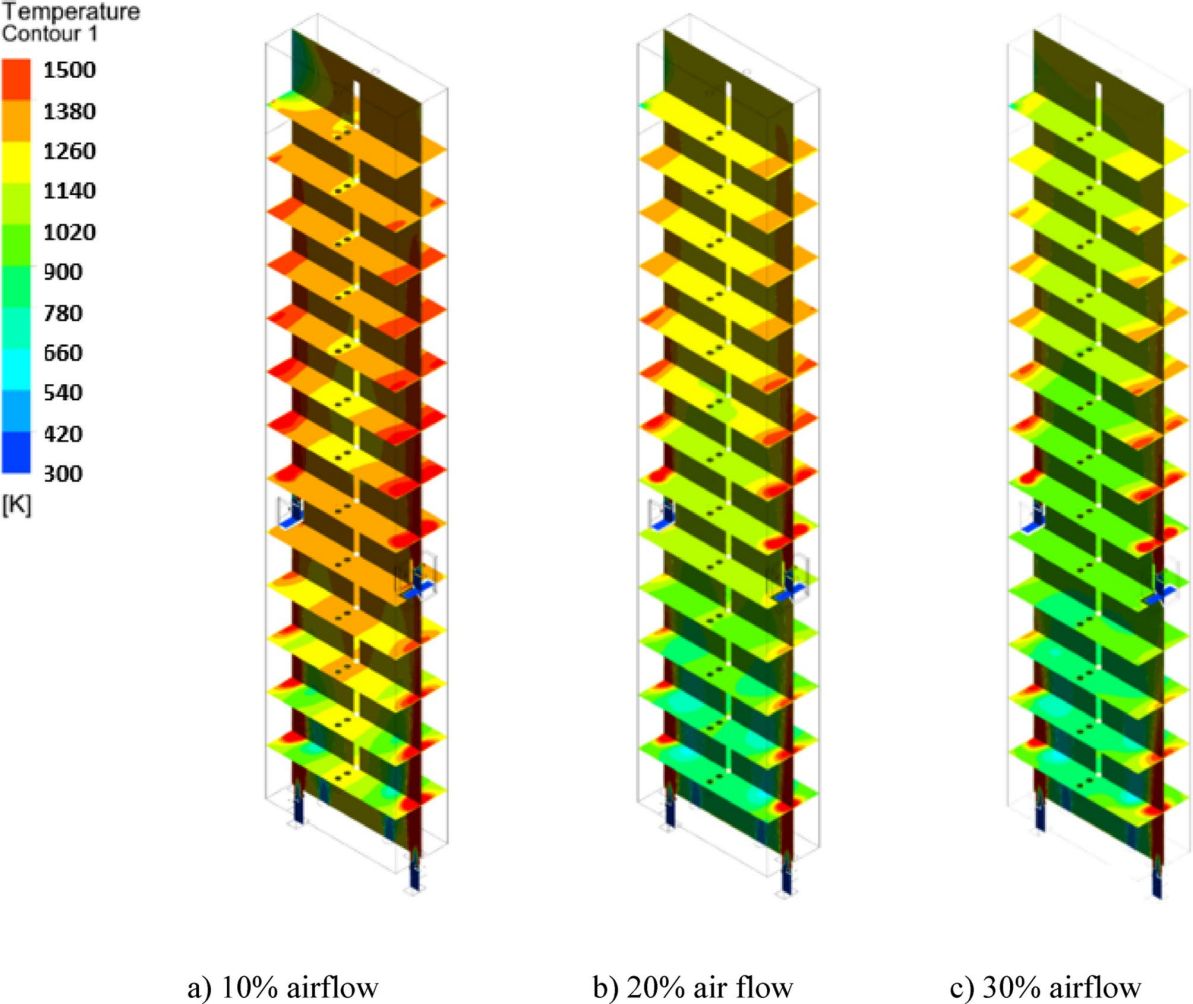
Effect of air flow on the temperature distribution in the furnace.

**Fig. 19:**
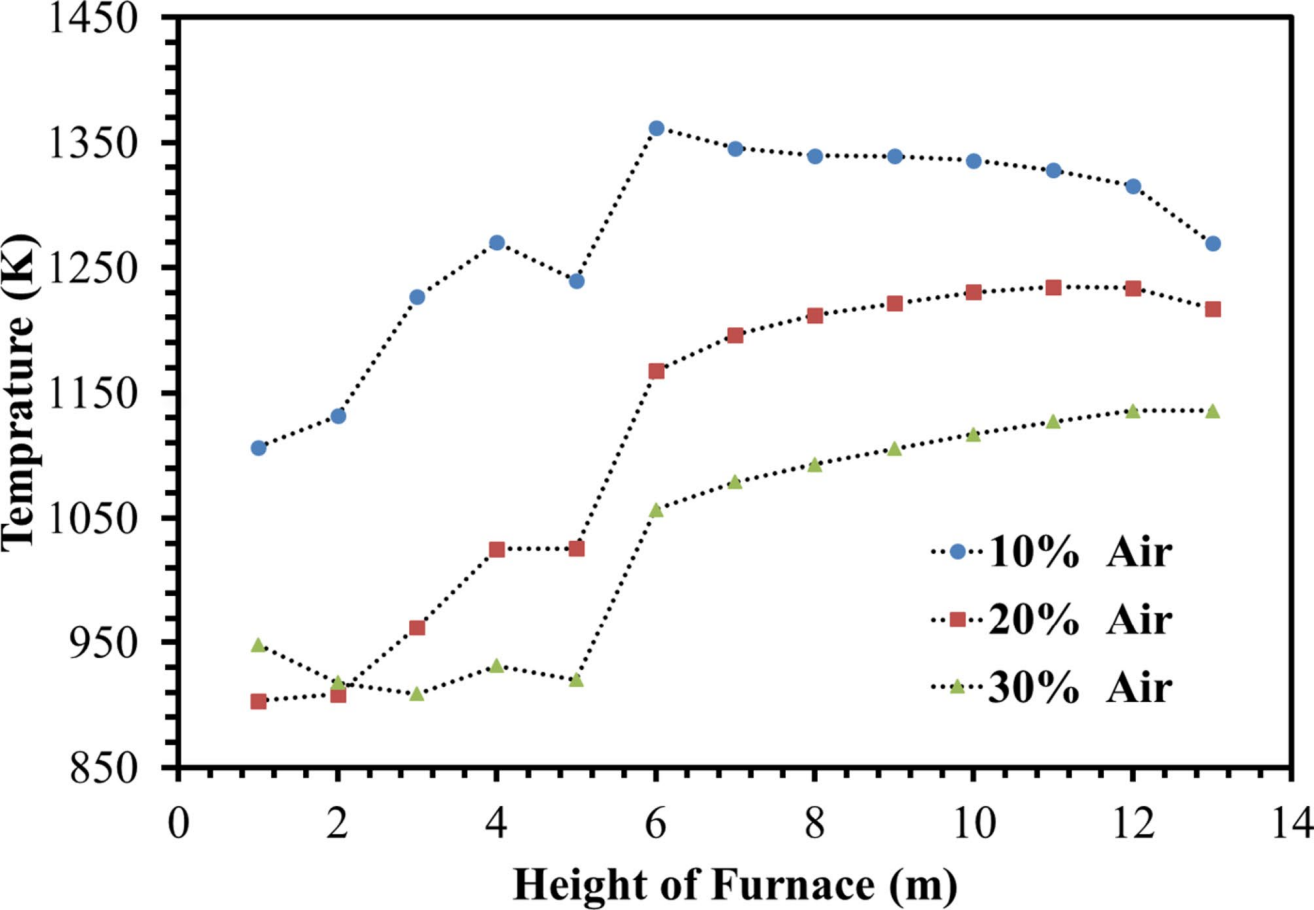
Effect of air flow on the average temperature profile in the furnace.

According to the results, increasing the temperature could not be only a good solution for complete coke burning. Moreover, increasing the residence time is also a very significant factor in coke burning. Therefore, a method has been introduced that would greatly increase the temperature of the furnace and the residence time of coke particles. In this idea, coke particles are injected separately into the furnace, along with ambient air at a different flow rate. The flow rates of the De-Coke stream were 10%, 20%, and 30% of the flow rate in operating conditions. Finally, the results were obtained as follows:

Figures 18 and 19 show temperature variations at different heights of the furnace. It is observed that injecting coke into the furnace with ambient air increases the temperature of the firebox. Thus, the average temperature of the combustion chamber for 10%, 20%, and 30% of the operation flow rate is 1277 K, 1118 K, and 1036 K respectively, therefore decreasing the amount of De-Coke stream caused to the increment of the combustion chamber temperature. In addition, since the heat produced inside the furnace is the same, the use of a lower flow rate of the incoming airflow along with the coke leads to an increase in the inside temperature of the furnace. Moreover, due to the presence of wall burners, the furnace inside temperature is showing an increasing trend.

**Fig. 20:**
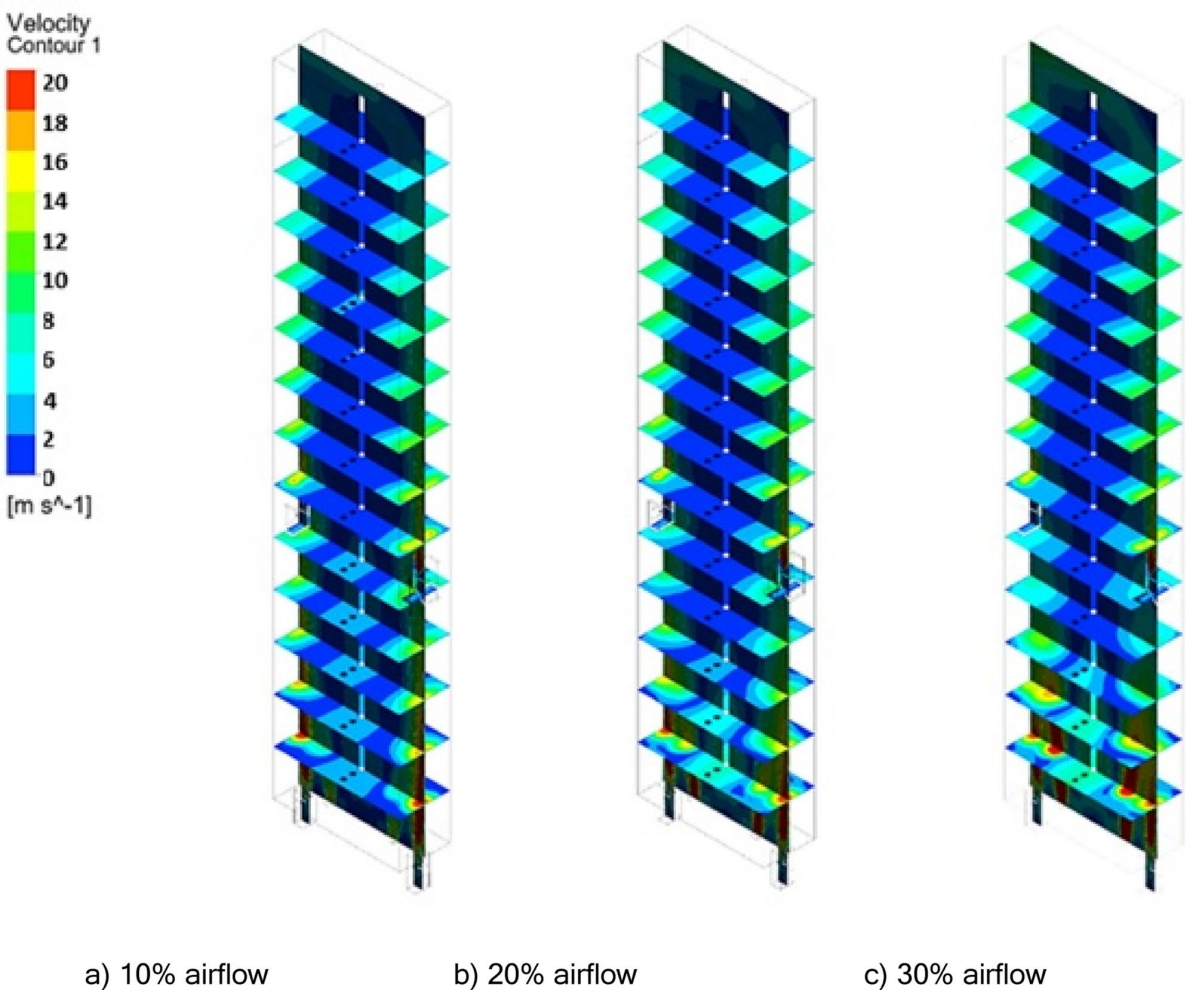
Effect of air flow on the velocity distribution in the furnace.

**Fig. 21:**
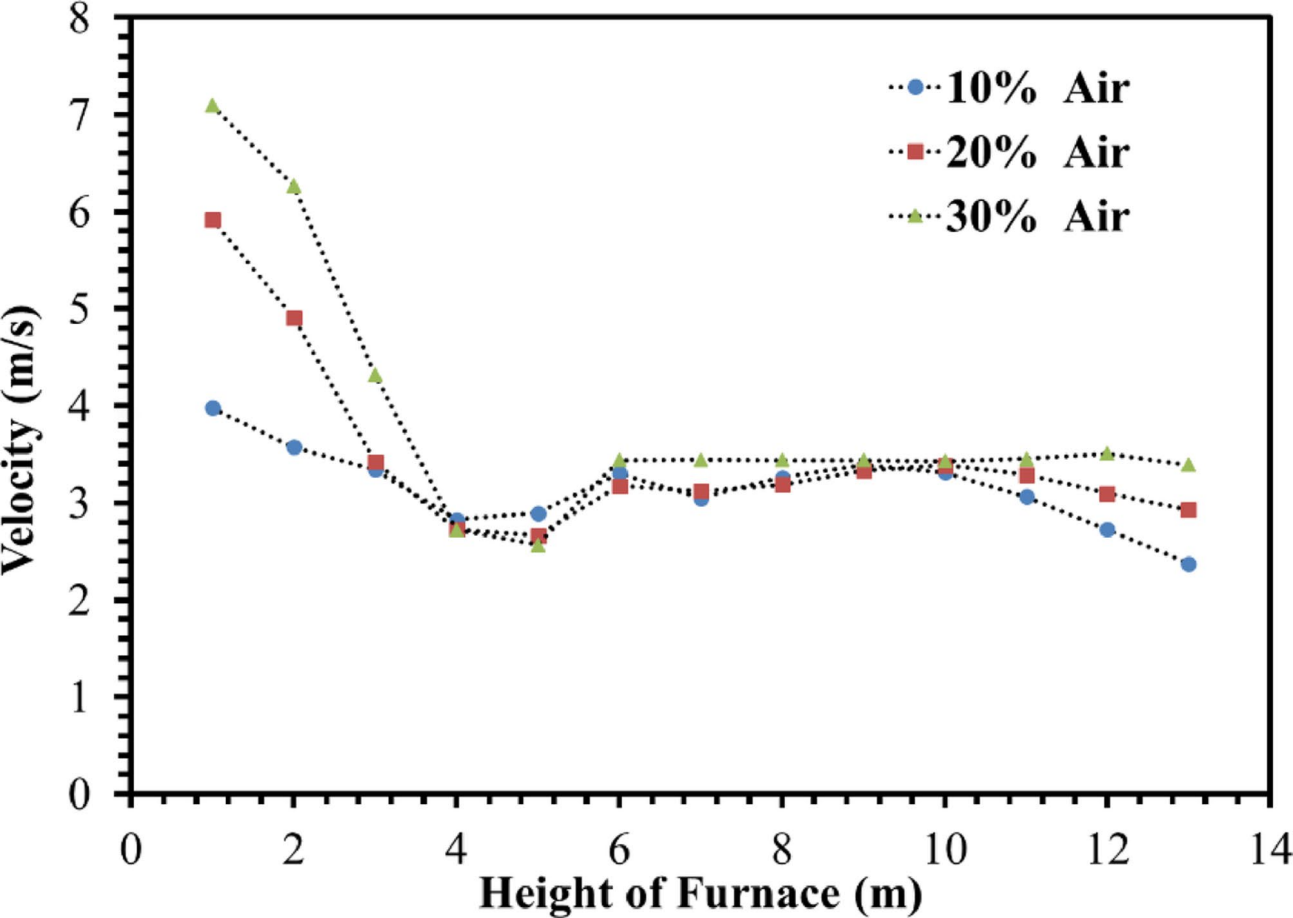
Effect of air flow on the velocity profile in the furnace.

Figures 20 and 21 show velocity variations at different heights of the furnace. It is observed that injecting coke into the furnace with ambient air increases the temperature of the firebox. So, the average velocity of the combustion chamber for 10%, 20%, and 30% of the operation flow is 3.16 m/s, 3.47 m/s, and 3.89 m/s respectively, therefore decreasing the amount of De-Coke stream caused the reduction in the combustion chamber gases velocity and thus caused to the increment to the residence time. There is a higher flow rate due to the injection flows at the bottom of the furnace. However, increasing the injection air flow rate has led to an increase in the velocity inside the furnace. This is even though after the height of about 4 m, the current is developed and its velocity becomes stable.

**Fig. 22:**
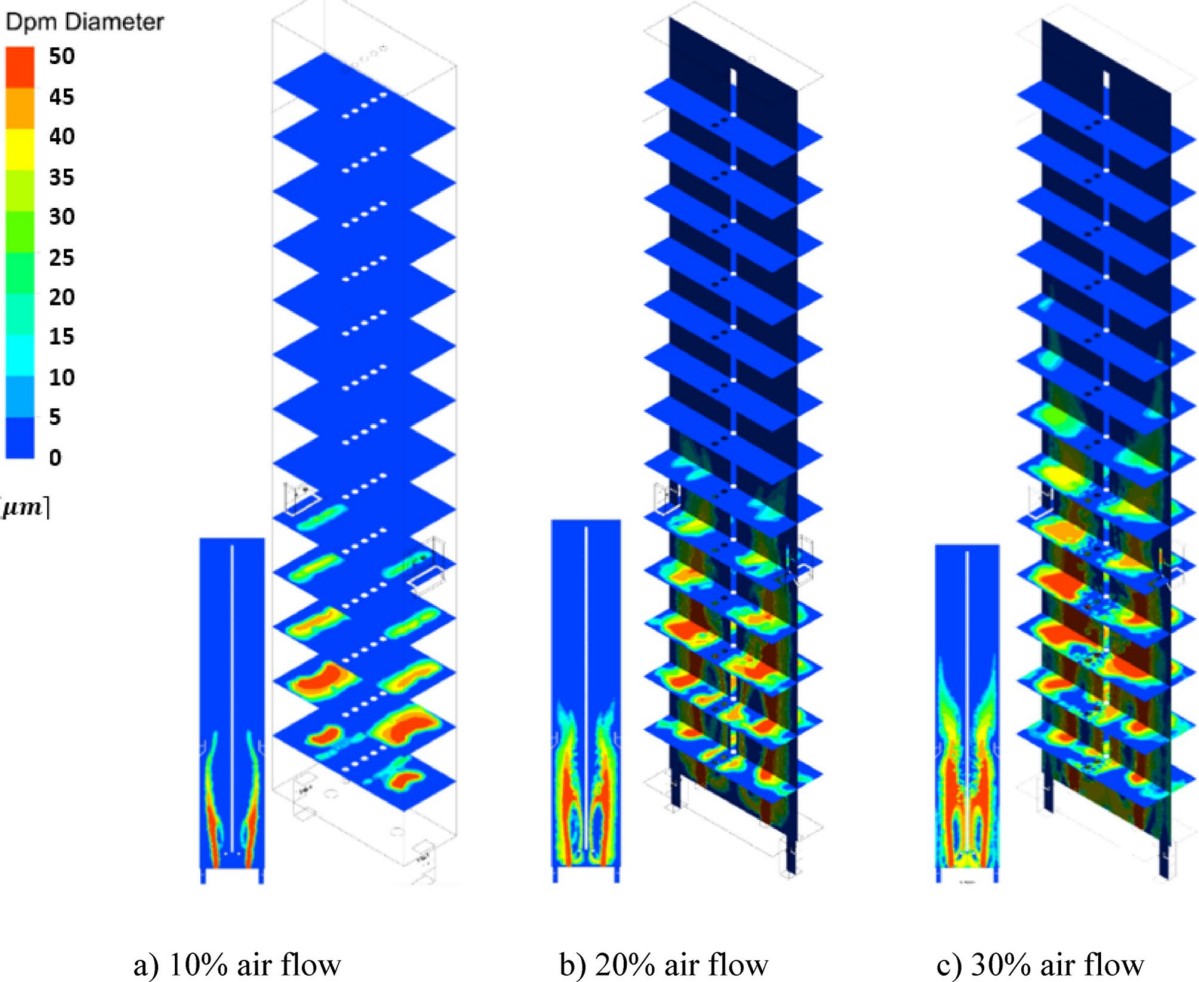
Effect of air flow on the particle diameter distribution in the furnace.

**Fig. 23:**
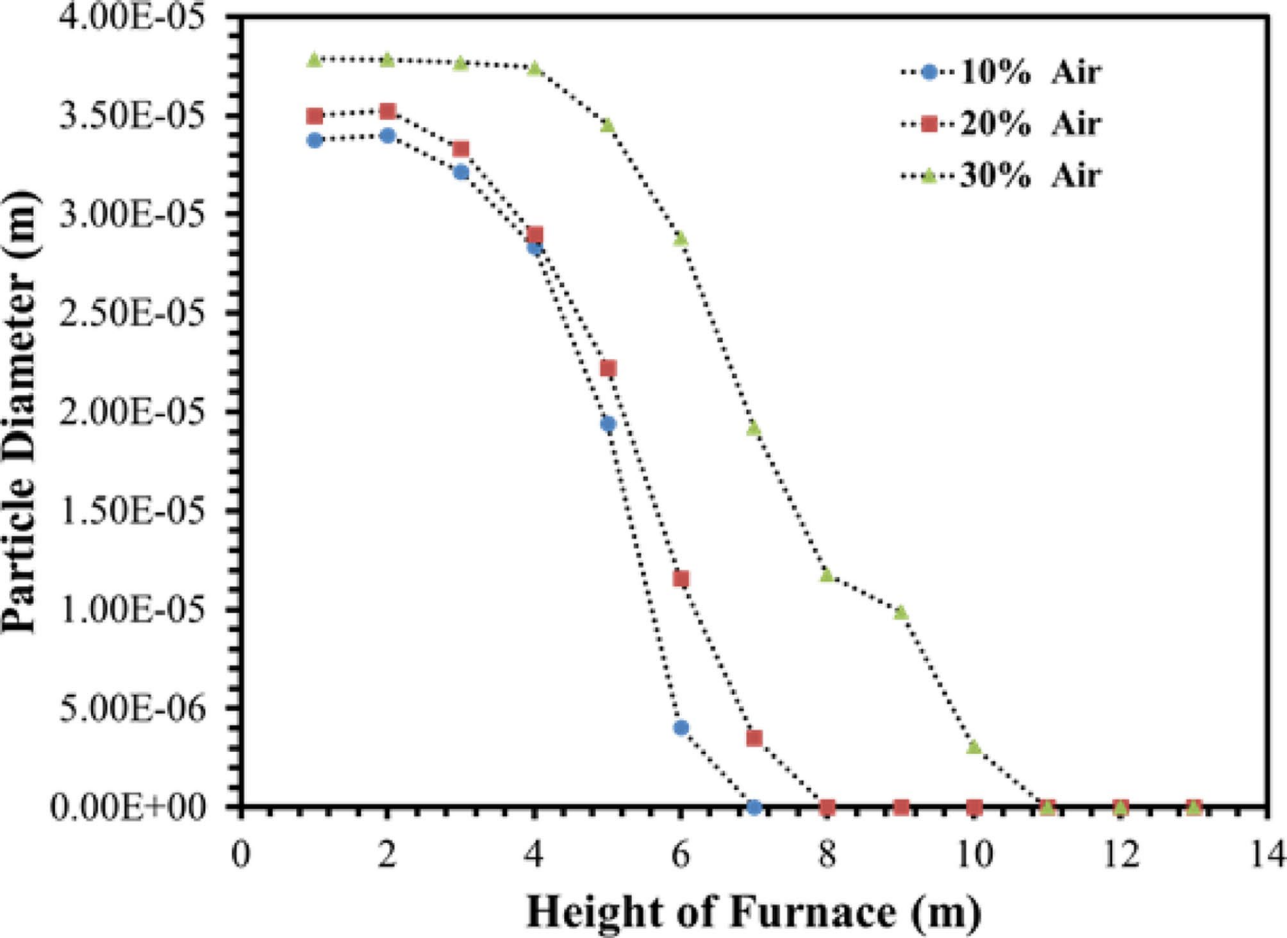
Effect of air flow on the particle diameter profile in the furnace.

Figure 22 shows the contours of the coke particle diameter distribution in the ISO-Surfaces (1 *m* distance from each other) along the y-direction. According to the shown contours, for the modes 10%, and 20% airflow from heights 6, and 7 m, and for the 30% airflow from a height of 11 m of the furnace there are no coke particles. At the mentioned heights, the coke particles are completely burned and destroyed in the furnace. Figure 23 shows the graph of the diameter of the coke particles injected into the combustion chamber at different heights of the furnace, in which the results were shown in Table 8. So, increasing flow of the De-Coke stream is a destructive factor for burning coke. In addition, a lower injection air flow rate leads to an increase in the temperature of the combustion chamber and a decrease in the gas flow rate, which results in an increase in the residence time and faster consumption of coke particles.

**Table 8:** Results for coke particles diameter in 10, 20 and 30% of operation flow.

Parameters	Diameter (m)	Maximum coke height (m)
10% of operation flow	2.53e − 5	6
20% of operation flow	2.77e − 5	7
30% of operation flow	3.57e − 5	11

**Fig. 24:**
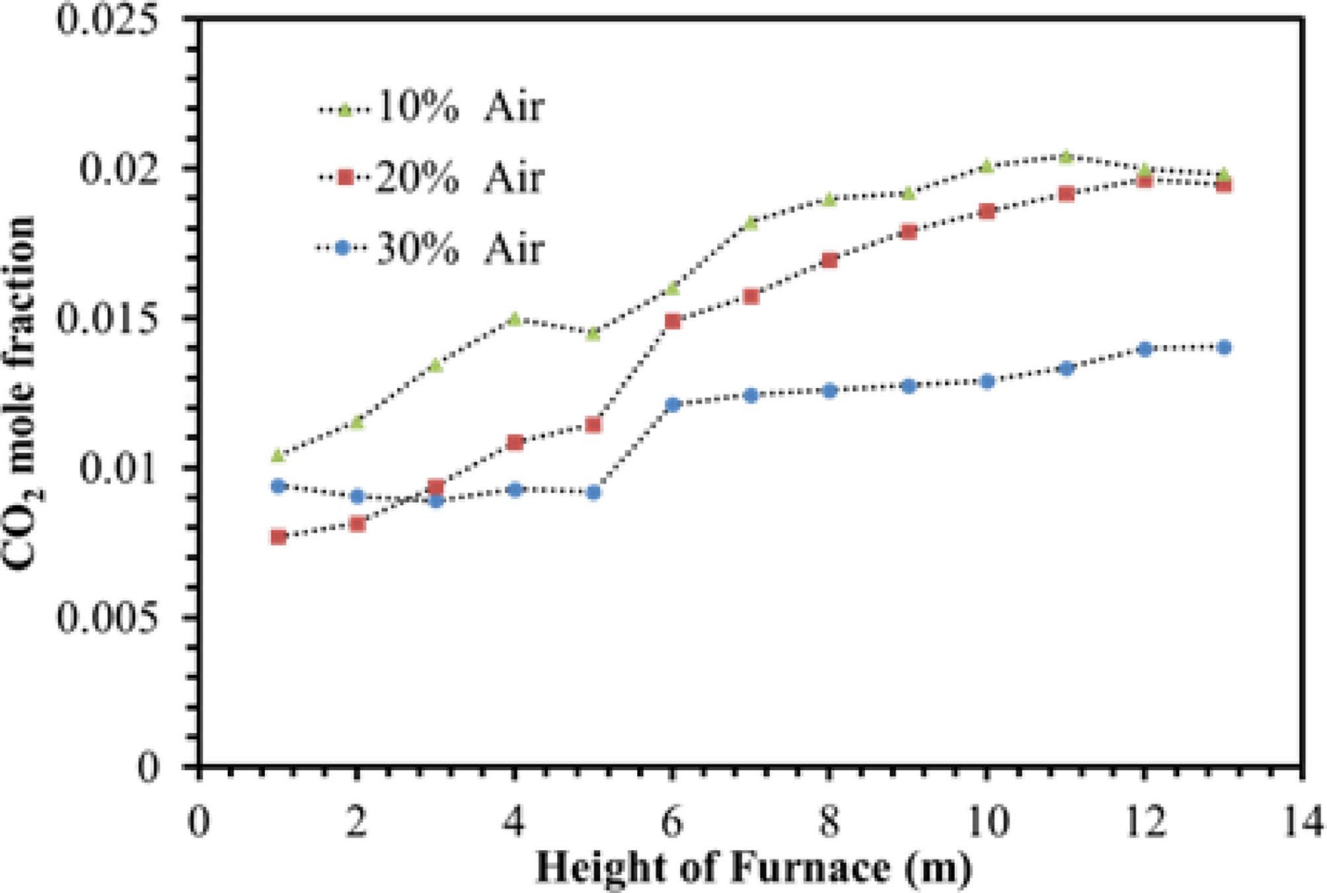
Effect of air flow on the CO2 mole fraction in the furnace.

Figure 24 shows the changes in carbon dioxide mole fraction in the furnace. It is showed that by air injection at the condition of 10%, 20%, and 30% modes of De-Cock flow, the average mole fraction of carbon dioxide in the firebox is 0.018, 0.014, and 0.011, respectively. It is the highest for the 10% air flow and the lowest for the state of 30%. Since with the decrease of the De-Coke flow, the temperature of the combustion chamber increased, and the conversion percentage of the particles increased, so it indicates more carbon dioxide composition. In all cases, it is explained that with more conversion of the coke at a higher altitude of the furnace, the concentration of carbon dioxide increases.

**Fig. 25:**
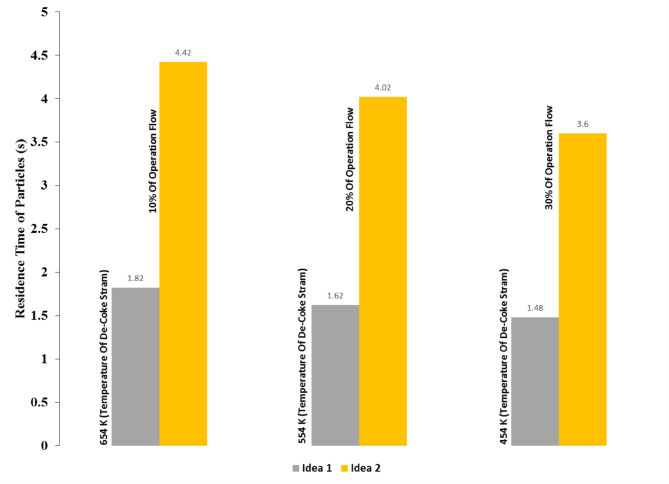
Comparison of particles residence time for ideas 1 and 2.

**Fig. 26:**
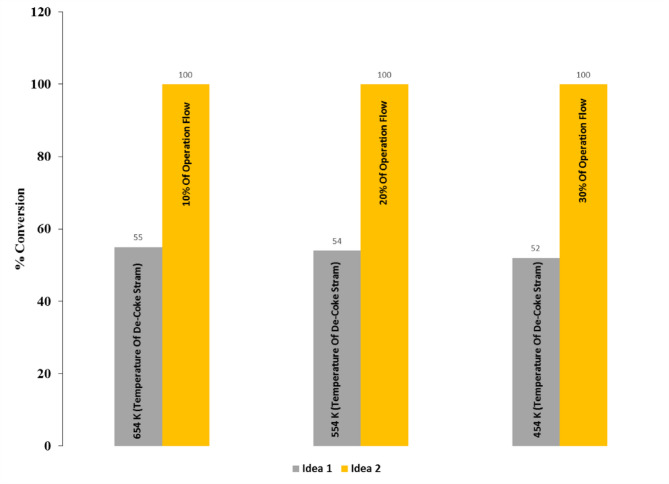
Comparison of conversion for ideas 1 and 2.

Figures 25 and 26 illustrate a comparison between two ideas that were studied in this investigation. These Figs show that residence time of coke particles is so important that has a significant effect on the coke conversion. Regarding numerical analysis via CFD method prove that Idea 1 which was increasing the De-Coke stream temperature before entering the combustion chamber, increased the temperature of the combustion chamber. When the De-Coke stream temperature increased from 454 K to 554 K, it was observed that the combustion chamber temperature increased from 773 K to 839 K, which means that increased by 66 degrees. This increase in gas temperature increased the velocity of the gases in the combustion chamber and thus reduced the residence time of the Coke particles as well as for cases in which the De-Coke stream temperature increased to 654 K, so the temperature of the combustion chamber from the initial state, which was 773 K, increased by 134 K and reached to 907 K. Finally, for both 554 K and 654 K, the conversion of Coke particles was 54% and 55% respectively.

Idea 2 unlike idea 1, is a batch process, in this idea, coke particles are collected and injected by a blower into the combustion chamber. From the results of this strategy, it was concluded that the coke particles rise a maximum to 11 m along the furnace. In this idea, the coke particles were injected into the furnace by a blower with three different flows, in which the percentage of conversion of coke particles to 100% was achieved in all three cases because the temperature of the combustion chamber increased by 504 K, as well as the residence time declined by 2.56 (s).

As can be seen from the results of Figs. 25 and 26, one can concluded that the optimal value for the rate of air injection beside the coke stream and temperature for the De-Coke stream, are 10% of the De-Coke flow and 654 K, respectively.

## Conclusion

This study numerically simulated an olefin furnace for ethylene production from ethane, focusing on the feasibility of injecting the De-Coke stream (containing water vapor, air, and coke particles) into the firebox to burn the coke particles. CFD was used alongside energy and exergy analyses. The results revealed that increasing the De-Coke stream temperature slightly improved coke conversion (from 52 to 55%) but reduced residence time due to increased gas velocity. Direct injection of coke particles by a blower achieved complete (100%) conversion by raising the combustion chamber temperature significantly and providing sufficient residence time. Energy and exergy analyses showed that in the De-Coke process at the coil, the highest energy and exergy were transferred as heat to the converter, while in the cracking process, most input energy went into the output products. Exhaust gas represented the largest energy loss across processes. The combustion furnace exhibited energy and exergy efficiencies of 44.8% and 29%, respectively, during cracking, with approximately 36% exergy destruction. In contrast, the cracking process at the coil achieved significantly higher efficiencies (93.24% energy and 96.2% exergy) and minimal exergy destruction (< 4%). Although coke combustion in the firebox reduces energy and exergy transfer to the coil and increases exergy destruction compared to conventional De-Coke, it enhances heat transfer and overall combustion efficiency. Managing exhaust gases from the furnace or firebox emerges as a key opportunity to improve olefin unit efficiency. Overall, the study highlights the importance of temperature, residence time, and energy-exergy management for optimizing coke combustion and furnace performance in olefin production.

## Data Availability

The datasets used during the current study are available from the corresponding author upon reasonable request.
